# Pediatric T-cell acute lymphoblastic leukemia blast signature and MRD associated immune environment changes defined by single cell transcriptomics analysis

**DOI:** 10.1038/s41598-023-39152-z

**Published:** 2023-08-02

**Authors:** Swati S. Bhasin, Beena E. Thomas, Ryan J. Summers, Debasree Sarkar, Hope Mumme, William Pilcher, Mohamed Emam, Sunil S. Raikar, Sunita I. Park, Sharon M. Castellino, Douglas K. Graham, Manoj K. Bhasin, Deborah DeRyckere

**Affiliations:** 1 Aflac Cancer and Blood Disorders Center, Children’s Healthcare of Atlanta, Atlanta, GA USA; 2 Department of Pediatrics, Emory University School of Medicine, Atlanta, GA USA; 3 Department of Biomedical Informatics, Emory University School of Medicine, Atlanta, GA USA; 4 Wallace H Coulter Department of Biomedical Engineering, Georgia Institute of Technology, Atlanta, GA USA; 5 Department of Pathology, Children’s Healthcare of Atlanta, Department of Pathology and Laboratory Medicine, Emory University School of Medicine, Atlanta, GA USA

**Keywords:** Cancer, Systems biology, Oncology

## Abstract

Different driver mutations and/or chromosomal aberrations and dysregulated signaling interactions between leukemia cells and the immune microenvironment have been implicated in the development of T-cell acute lymphoblastic leukemia (T-ALL). To better understand changes in the bone marrow microenvironment and signaling pathways in pediatric T-ALL, bone marrows collected at diagnosis (Dx) and end of induction therapy (EOI) from 11 patients at a single center were profiled by single cell transcriptomics (10 Dx, 5 paired EOI, 1 relapse). T-ALL blasts were identified by comparison with healthy bone marrow cells. T-ALL blast-associated gene signature included *SOX4, STMN1, JUN, HES4, CDK6, ARMH1* among the most significantly overexpressed genes, some of which are associated with poor prognosis in children with T-ALL. Transcriptome profiles of the blast cells exhibited significant inter-patient heterogeneity. Post induction therapy expression profiles of the immune cells revealed significant changes. Residual blast cells in MRD+ EOI samples exhibited significant upregulation (*P* < 0.01) of PD-1 and RhoGDI signaling pathways. Differences in cellular communication were noted in the presence of residual disease in T cell and hematopoietic stem cell compartments in the bone marrow. Together, these studies generate new insights and expand our understanding of the bone marrow landscape in pediatric T-ALL.

## Introduction

Pediatric T-cell acute lymphoblastic leukemia (T-ALL) is caused by abnormal proliferation of immature T cells in the bone marrow microenvironment (BME) and accounts for approximately 15% of childhood leukemias. T-ALLs express T-cell markers including CD3D, CD2, CD4, CD5, CD7 and CD99 on their cell surface^1^ and flow cytometric analysis of cell surface markers enables T-ALL classification based on the stage of differentiation of leukemic clones into pro-T, pre-T, cortical and mature T-ALL. Additionally, early T-cell precursor (ETP) ALL, is a specific subtype of T-ALL with similarities to T/myeloid mixed phenotype acute leukemia that has been associated with a higher risk for induction failure and relapse by some groups^2,3^. While outcomes for children and adolescents with T-ALL have steadily improved and event free survival (EFS) rates are now more than 85% using contemporary therapeutic approaches, treatment of specific high-risk subsets or in the setting of relapsed disease remains challenging^3^.

Advancements in next generation sequencing (NGS) methods have propelled studies investigating the mutational landscape of T-ALL. These studies have implicated a spectrum of oncogenic insults including activating mutations (e.g., *NOTCH1*, chromosomal rearrangements involving transcription factors such as *HOXA*, *TAL1*, *TLX1*, fusion genes such as tyrosine kinase *ABL1*), deletions in tumor suppressor genes and cell cycle inhibitors (e.g. *CDKN2A*), and mutations in epigenetic regulators and chromatin modifiers^4–6^. *NOTCH1* activating mutations or impaired degradation of NOTCH1 resulting from *FBXW7* mutations are frequent in T-ALL^7^ and often accompanied with *CDKN2A* deletions. These studies highlight the variability of molecular insults associated with T-ALL. Also, the prevalence of different mutations varies between pediatric and adult T-ALL as does the response to treatment. The heterogeneity of the mutations complicates their prognostic relevance in T-ALL^6^. The presence or absence of small numbers of persistent leukemic blasts in the marrow at the end of induction (EOI) therapy, termed minimal residual disease (MRD), remains the most reliable measure of response to therapy in T-ALL. MRD is assessed at the EOI and consolidation phases of treatment generally by flow cytometry using markers that can detect abnormal cells at frequencies  ≤ 0.01%. MRD negativity is associated with favorable outcomes in T-ALL^8–10^ and is used for risk stratification and to guide the course of treatment. Relapsed disease has been attributed to the selection and proliferation of subclones carrying mutations that confer therapy resistance and disease progression^11^. Recurrent disease is treated with intensive therapy and hematopoietic stem cell transplant, and yet, prognosis remains dismal^3^.

Analysis of the interactions between leukemia cells and host cells in the BME that support disease progression or therapy resistance has also provided critical insights into leukemia biology. The BME is comprised of stromal and immune cells such as lymphocytes and monocytes, as well as blood vessels. Tumor cells interact with the BME through membrane receptors, growth factors and cytokines to promote a tumor nurturing environment accompanied by enhanced vasculature to counter hypoxic conditions^12,13^. Aberrant interactions manifest in dysregulated signaling in networks such as PI3K/AKT/mTOR, MEK/ERK, and protein kinase C (PKC) in T-ALL^14–16^. The diverse spectrum of genomic aberrations in T-ALL underscores a common theme of dysregulated pathways leading to uncontrolled proliferation of leukemic cells by both cell autonomous mechanisms and interactions with the BME. These networks present opportunities for therapeutic targeting. For instance, disruption of CXCL12-CXCR4 signaling between bone marrow (BM) stromal cells and leukemic blasts impaired disease progression in murine models, implicating strategies to block interactions that support a tumor permissive environment as a promising therapeutic approach^17^. Likewise, NOTCH activating mutations accompanying T-ALL can upregulate IL-7 receptor, which provides a survival advantage to leukemia cells^18,19^. Inhibition of hypoxia-induced HIF1α signaling can also promote leukemia progression by helping cells adapt to hypoxic conditions in the BM environment and promoting chemoresistance^20,21^.

The path towards attaining complete remission (CR) involves understanding the molecular profiles associated with response/failure to standard therapy, which can then be applied to inform novel intervention strategies^22^. Recently, single cell RNA sequencing (scRNA-Seq) has been used to dissect the molecular interactions in multiple hematologic malignancies. van Galen et al., utilized single cell sequencing of BM aspirates from adult patients with acute myeloid leukemia (AML) to study how different cell types contribute to disease progression^23^. Bailur and colleagues provided evidence of immune exhaustion and dysfunction in the BME in pediatric patients with B-ALL or AML using mass cytometry and single cell genomics^24^. In the case of T-ALL, clonal heterogeneity and acquisition of mutations has been studied by targeted single cell and single cell DNA amplicon sequencing^25,26^. In an ETP-ALL study, Anand and colleagues utilized full-length single cell RNA sequencing to study stem-like states that mediate resistance by modulating the immune environment^27^. The unique insights provided by these studies affirm the power of single cell approaches to better comprehend the intricate BME landscape by dissecting the roles of both leukemia and BME cells in T-ALL pathogenesis. Toward this end, we performed single cell transcriptome analysis on pediatric T-ALL BM samples collected at diagnosis (Dx), EOI and relapse. Cell populations, their signaling interactions and dysregulated pathways in different cell types were analyzed to develop a comprehensive map of the BME landscape before and after induction therapy. We also studied the interactions between different BME cell types in MRD +  and MRD− samples to generate first insights into differences in cell communication patterns based on response to induction therapy.

## Results

### Single cell transcriptome profiling reveals enriched heterogenous T-ALL blasts and few immune cell clusters at time of diagnosis

**Figure1:**
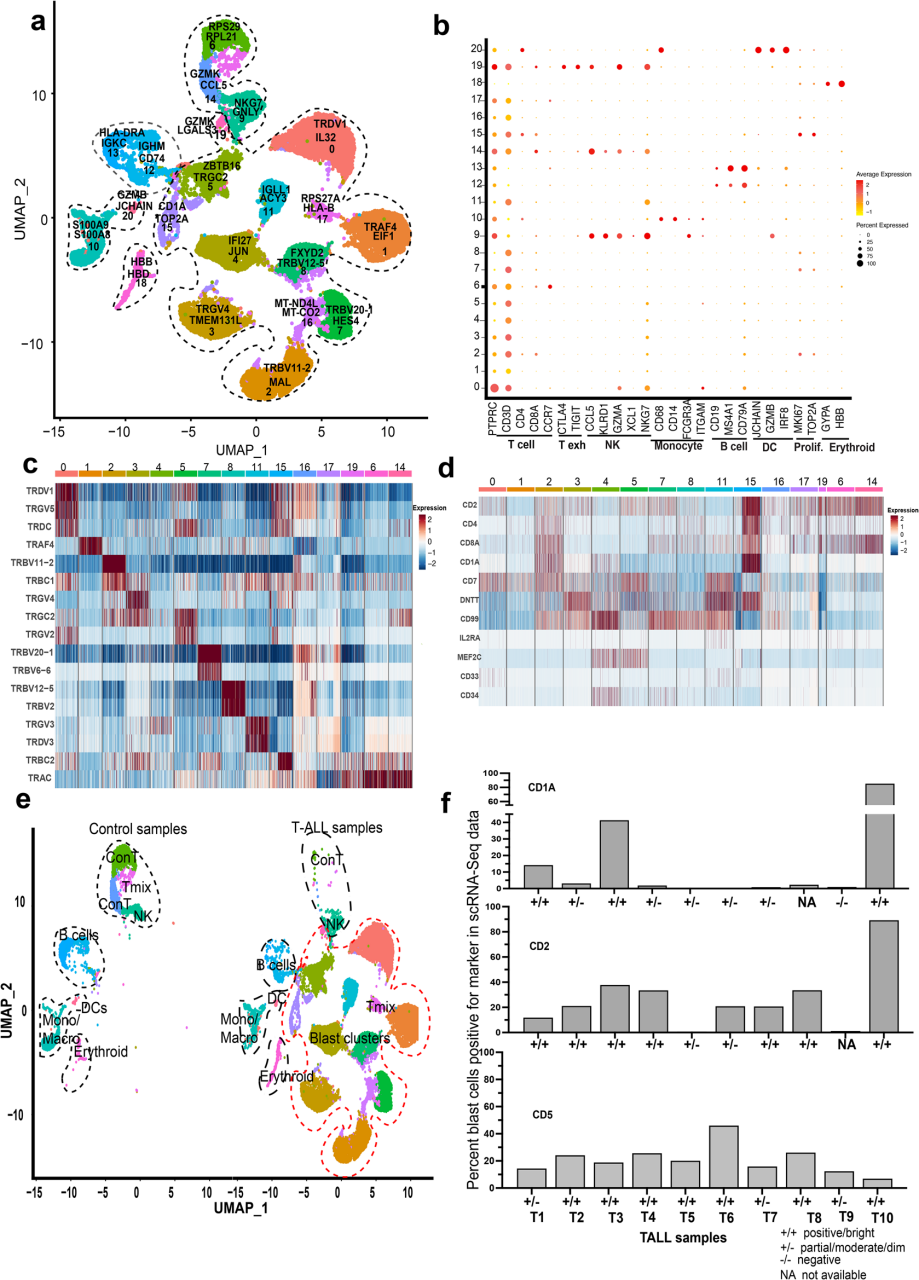
ScRNA-seq profile of pediatric T-ALL bone marrow at diagnosis. (**a**) Combined UMAP of 10 pediatric T-ALL and four healthy control BM samples showing cells in 21 different clusters. Each cluster is uniquely colored and designated with a cluster number. Two overexpressed gene markers are also shown with each cluster. (**b**) Dot plot showing annotation of the cell clusters based on expression of known gene markers. The clusters are numbered along the y-axis and x-axis denotes the markers for T cells, T exhausted, NK, monocytes/macrophages, B cells, DCs, proliferating and erythroid cells. (**c**) Heatmap (downsampled randomly to 131 cells per cluster) showing scaled expression of different TCR isoforms in the CD3D +  clusters with red and blue colors denoting upregulation and downregulation respectively. (**d**) Heatmap (downsampled randomly to 500 cells per cluster except cluster #19) showing scaled expression of T-ALL markers in the CD3D +  clusters with red and blue colors denoting upregulation and downregulation respectively. (**e**) Split UMAP plot showing cluster-wise distribution of healthy control bone marrow cells on the left and pediatric T-ALL patient bone marrow cells on the right. (**f**) Bar plots showing single cell expression data for percent of blast cells (y-axis) expressing the indicated markers (CD1A, CD2, CD5) in each patient bone marrow sample (x-axis) at diagnosis. + / + , + /−, −/−, and NA represent flow cytometric surface protein designation as positive/bright, partial/moderate/dim, negative and data not available, respectively.

Single cell transcriptome profiling of 10 Dx T-ALL pediatric BM samples (T1-10) was performed. The clinical information of the samples used in this study has been summarized in supplementary Table S1. To determine known cell types, we included four healthy BM samples from a recent single cell study^24^. Figure 1a shows a combined UMAP of 24,852 cells from 10 diagnostic pediatric T-ALL samples and 5,878 cells from the four healthy control BM samples. The cells separated into 21 transcriptionally distinct clusters. Each of the clusters is designated by a cluster number and two of the top cluster overexpressed gene markers determined by the ‘Find AllMarkers’ function (Fig. 1a). The cells were annotated based on expression of known gene markers into T cells, B cells, monocytes/macrophages, natural killer (NK) cells, dendritic cells (DCs), and erythroid cell clusters (Fig. 1b). We noted that majority of the cells (> 75%) in the clusters 0–8,11, 14–17 and 19 were positive for *CD3D* expression. The top cluster distinguishing markers (Table S2) also revealed overexpression of different TCR isoforms in several of the *CD3D* +  clusters as shown in the heatmap in Fig. 1c. For clarity, the heatmap was downsampled randomly to visualize equal number of cells from each cluster based on the number of cells in cluster 19, the smallest *CD3D* +  cluster. We then examined the expression of known T-ALL markers in the *CD3D* +  clusters to evaluate blast marker expression. The heatmap (downsampled) in Fig. 1d shows expression of several T-ALL related markers in these clusters. Further, as shown in the split UMAP plot of the Dx T-ALL and healthy control samples (Fig. 1e), some of the cell clusters (NK, B, Erythroid, Monocyte/macrophage clusters) contained cells from both the control and T-ALL samples, while two of the *CD3D* +  clusters, 6 and 14, were enriched mostly in controls (Con T). Most of the other *CD3D* +  clusters, enriched in TCR isoforms, and positive for T-ALL marker expression comprised of cells from the T-ALL samples only. Cluster 17 comprised of cells from control and T-ALL samples, was designated as Tmix. Based on these results and the BM blast percentages available from clinical records (Table S1), the clusters 0–5,7,8,11,15,16,19 were designated as blast clusters.

### Gene expression data correlates with patient sample immunophenotype

T-ALL immunophenotypes determined by standard flow cytometry based semi-quantitative analyses (i.e., ‘bright/positive’, ‘partial’, or ‘dim/negative’) were obtained from clinical records and compared with single cell-based quantification of marker genes to determine concordance in the two techniques since the former is largely based on surface protein expression while the latter is transcriptome based. We computed the percent of blast cells positive for selected marker expression based on single cell data. Figure 1f and supplementary Fig. S1 show the percent cells expressing various T-ALL cluster markers determined by scRNA-Seq and the corresponding flow cytometry-based designation as positive, partial, or negative, respectively. In most cases where samples were scored as bright/positive for a given marker, the single cell expression and flow cytometry data correlated well. However, there was some inconsistency between immunophenotyping designation and scRNA-Seq measurement for markers where expression was very low in the scRNA-Seq assay (e.g., *CD58, TDT*) and the immunophenotyping designation was determined to be partial/negative.

### T-ALL blast transcriptomes exhibit significant heterogeneity between patients

**Figure 2:**
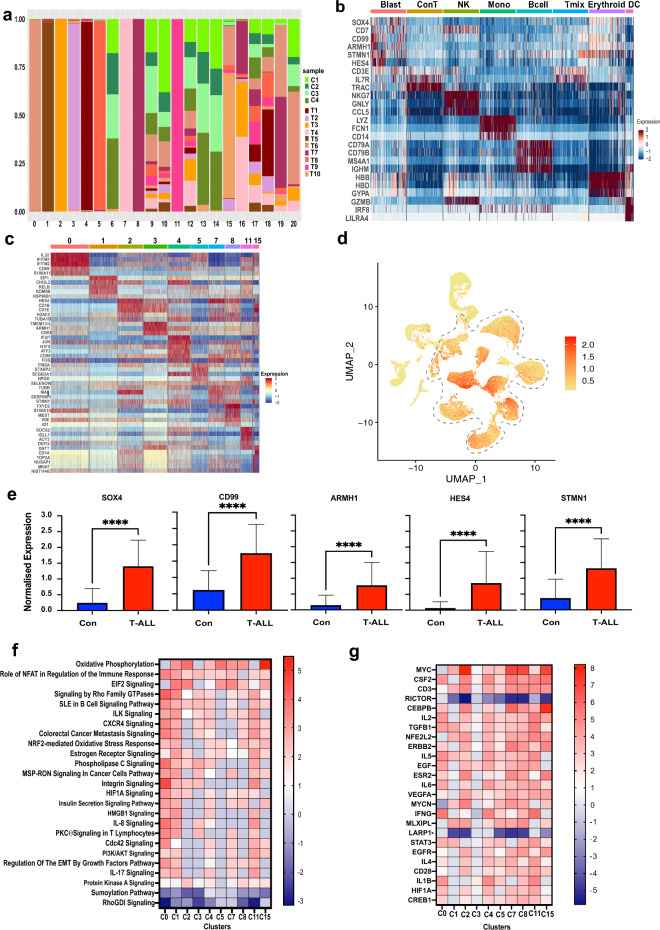
Pediatric T-ALL bone marrow blast expression profiles. (**a**) Bar plot showing sample-wise percent composition of each cluster in the merged Dx T-ALL and control samples Seurat object as indicated by the legend. (**b**) Heatmap (downsampled randomly to 500 cells each cell type except DCs) showing scaled expression of canonical markers for different cell types across annotated clusters. Red and blue indicate upregulation and downregulation, respectively. (**c**) Heatmap showing scaled expression of select top cluster distinguishing genes in majority patient specific blast clusters with cluster numbers on the top. (**d**) Feature plot of the merged Dx T-ALL and control samples showing combined expression of genes *SOX4, JUN, STMN1, CD99, TUBA1B*. (**e**) Relative expression of the indicated markers in control BM samples (blue) vs pediatric T-ALL samples (red). The asterisk denotes significance (*P* < 0.0001). (**f**) Heatmap showing pathways commonly dysregulated in different T-ALL patient blast clusters using the IPA analysis tool (Qiagen). The color scale on the right represents the Z-score indicating the extent of upregulation (red) or downregulation (blue). (**g**) Upstream regulatory molecules significantly activated (red) or inhibited (blue), as denoted by the color scale (Z-score) to the right, in the different T-ALL patient blast clusters (x-axis).

To understand the cellular complexity of the T-ALL landscape, we looked at the cluster-wise distributions of the patient Dx samples (T1-10). Cluster-wise distribution analysis of the T-ALL samples showed that many of the clusters (clusters 0–5, 7, 8, 11) are predominantly made up of > 95% cells from individual patient samples (Fig. 2a). Clusters 15 and 19 each comprised cells from two T-ALL samples while cluster 16 mostly comprised of cells from 3 T-ALL samples. These clusters showing expression of blast-associated markers were commonly annotated as Dx blast clusters while the rest of the clusters show expression of canonical cell type specific markers: T cells (enriched in the healthy controls; *CD3E*^+^, *IL7R*^+^), NK cells (*NKG7*^+^), monocytes (*FCN1*^+^), B cells (*CD79A*^+^ and *CD79B*^+^), erythroid cells (*HBB*^+^), and DCs (*LILRA4*^+^) as shown in a downsampled heatmap in Fig. 2b. The differentiated cell clusters were comprised of cells from multiple patients, unlike the patient specificity seen with the majority of blast cell clusters. The top differentially expressed genes in the patient blast clusters include interferon induced transmembrane *IFITM* family members, *CD1* family members, NOTCH1 target gene *HES4*, *TOP2A* topoisomerase, *JUN* and *FOS* oncogenes, and the gene for myelin and lymphocyte protein *MAL*. These genes that are overexpressed in select patient specific clusters are also expressed to varying levels in the blast clusters from some of the other patients, indicating that they are commonly dysregulated. Figure 2c shows a heat map of select top markers from the majority patient specific blast clusters. The most differentiating genes in these clusters also included the TCR isoforms (Fig. 1c**, **Table S2).

### Gene expression signature of T-ALL blasts

We performed comparative analysis of the Dx blast microenvironment clusters relative to the control T cell clusters (clusters 6 and 14 in Fig. 1a) to identify a commonly dysregulated gene signature associated with the T-ALL blasts (patients T1-10 at Dx). The differential expression analysis identified 119 genes that are significantly overexpressed (*P* value < 0.05), based on 1.5-fold change cutoff, in blast cells (Tables 1, S3). Some of the significantly overexpressed gene markers in T-ALL blasts have already been associated with T-ALL, such as *CD99* and *DNTT*^28^. Others genes like *SOX4, CDK6* have been shown to be associated with T-ALL proliferation^29,30^. Similarly, *HES4*, is a known target gene of the T-ALL driver, NOTCH1^31^. Contrary to this, not much is known about genes such as *NME2, CHI3L2, SELENOW* and *ARMH1* in the context of pediatric T-ALL. This suggests a broader dysregulated network of genes persisting across heterogenous blast clusters from different patients. Combined expression of the top genes *SOX4, JUN, STMN1, CD99,* and *TUBA1B* from this blast signature was enriched in T-ALL blast cell clusters compared to other cells (Fig. 2d). Expression of the genes from this signature was also significantly higher in T-ALL samples relative to healthy controls. Figure 2e shows comparison of overall expression (across all cell types) of select blast associated genes (*SOX4, STMN1, CD99, HES4, ARMH1)* in T-ALL BM samples relative to healthy controls indicating that the elevated expression of these genes should be discernible in bulk samples as well. Further, to explore the association of the top genes from this signature with event free survival (EFS) outcome, we performed validation and survival analysis using the gene expression, EFS, MRD and CNS stage at diagnosis data from the TARGET ALL phase 2 (T-ALL) cohort^32^. Combined overexpression of the top 25 genes (Table 1) from this signature was significantly associated with poorer EFS (HR = 2.7, LR *P* value = 0.008) (Fig. S2). Table S4 summarizes the hazard ratios from the univariate and multivariate analysis. The multivariate analysis based on gene expression, CNS, and MRD status depicted a significant association of multiple genes from this signature with poor outcome including *STMN1, NME2, CDK6, IER2* and *ARMH1*. The univariate and multivariate analysis both depicted a consistently significant association of *CDK6* and *ARMH1* genes from this signature with poor outcomes. The event-free survival analysis based on MRD status alone depicted a non-significant association with outcome whereas CNS stage 3 depicted a significant association with poorer outcome.

**Table 1:** Top 25 genes significantly overexpressed in T-ALL blasts.

	gene	avg_log2FC	pct.1	pct.2
1	MT-ND4L	3.65	0.99	0.49
2	MT-ATP8	3.10	0.97	0.18
3	SOX4	2.35	0.91	0.12
4	JUN	2.27	0.82	0.26
5	STMN1	2.23	0.86	0.24
6	TUBA1B	2.19	0.82	0.43
7	CD99	2.16	0.96	0.53
8	KLF6	2.13	0.86	0.25
9	HES4	2.11	0.54	0.02
10	FOS	2.02	0.62	0.16
11	EEF1G	2.02	0.93	0.27
12	TUBB	1.85	0.84	0.38
13	TUBA1A	1.80	0.85	0.25
14	PTPRCAP	1.74	0.75	0.02
15	GSTP1	1.66	0.85	0.34
16	MZB1	1.60	0.68	0.03
17	NME2	1.60	0.76	0.07
18	PPP1R15A	1.47	0.70	0.12
19	TAGLN2	1.47	0.89	0.44
20	CHI3L2	1.43	0.41	0.04
21	CDK6	1.43	0.68	0.07
22	SELENOW	1.43	0.81	0.35
23	GADD45B	1.37	0.57	0.17
24	IER2	1.34	0.79	0.45
25	ARMH1	1.33	0.67	0.16

We also evaluated gene signatures from individual patient-specific blast clusters to analyze the key pathways and regulators using the Ingenuity Pathway Analysis tool (IPA, Qiagen). The comparative analysis identified pathways (Fig. 2f) and regulators (Fig. 2g) that are most dysregulated among the patient-specific clusters. Oxidative phosphorylation was commonly upregulated in most blast clusters and pathways associated with oncogenic signaling e.g. EIF2 signaling, PI3/AKT, and HIF1A are upregulated in many clusters. Integrin Linked Kinase (ILK) signaling which has been implicated in the survival of leukemic cells in the BM^33^, and Integrin signaling are also increased. Among the regulators, CSF, MYC, CD3, and TGFB1 are commonly upregulated while RICTOR and LARP1 are commonly downregulated. These analyses highlight the gene-signatures, pathways and key transcriptional regulators commonly associated with malignant phenotypes and open avenues for identification of pan T-ALL therapeutic targets.

### Comparative analysis of paired samples reveals presence of residual blast cells after treatment

**Figure 3:**
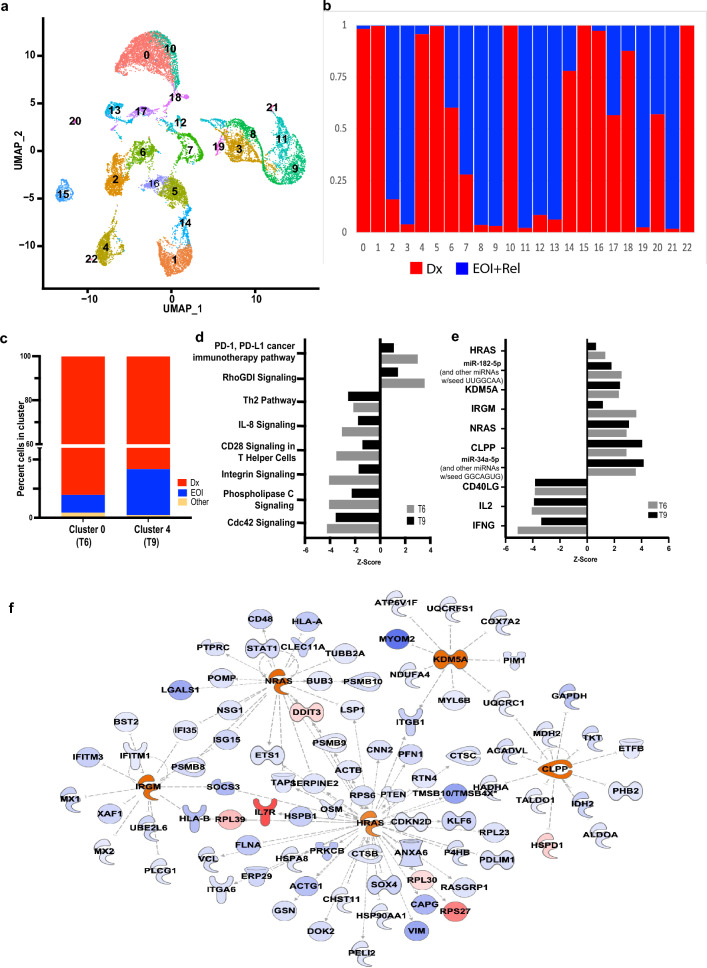
Investigation of residual blast cells in EOI samples. (**a**) UMAP plot showing BM cells from 5 paired Dx and EOI samples (patients T6-T10) and one unpaired relapse sample (patient T11) in 22 transcriptionally distinct clusters. (**b**) Bar plot showing sample-wise percent composition of each cluster in the merged Seurat object from Fig. 3a, with Dx samples indicated in red and EOI and relapse samples indicated together in blue. (**c**) Bar plot showing percent composition of clusters 0 (predominantly comprised of cells from sample T6) and 4 (predominantly comprised of cells from sample T9) with Dx and EOI cells coming from the same sample time points denoted in red and blue, respectively, and cells from the other patient samples in that cluster shown combined in yellow color. (**d**) and (**e**) show commonly dysregulated pathways and regulators, respectively, in residual cells at EOI from samples T6 and T9 identified using the IPA tool, from the differential expression analysis between the paired EOI and Dx cells. (**f**) Map of upstream regulators and their interactants in residual blast cells. Red/blue denote extent of upregulation and downregulation, respectively.

The goal of induction therapy is to achieve remission by successful clearance of leukemia blast cells. Transcriptome profiling of paired Dx and EOI pediatric BM samples enabled us to probe how induction treatment alters the BM cellular transcriptome. Figure 3a shows a combined UMAP of 18,373 cells from 5 Dx samples (patients T6-T10), 5 paired EOI samples and one unpaired relapse sample (patient T11). The UMAP and clustering analysis formed 22 transcriptionally distinct clusters. Figure 3b shows the comparative cell proportions from Dx (red) and EOI + Relapse (blue) samples in individual clusters. The presence of predominant Dx or EOI + Relapse specific clusters reveals a significant shift in the makeup of the cellular microenvironment post treatment. Analysis of the signature genes derived from the blast cells at Dx revealed that the expression of these genes was diminished in the EOI and relapse cells (Fig. S3). It is evident from the scRNA-Seq profiles that at EOI most of the leukemic blast cells are eliminated. While most of the blast cell clusters were almost entirely made up from Dx cells, there were a few clusters (e.g., clusters 0, 4) that had a small fraction of cells at EOI that clustered with Dx blast cells. We examined these clusters to see if they comprised cells from a Dx -EOI pair, i.e., pre and post treatment samples from the same patient (Fig. S4). The clusters 0 and 4, each comprised of paired samples with greater than 95% cells from Dx sample and a very small fraction of cells from the matched EOI sample (Fig. 3c). Cluster 0 comprised Dx and EOI cells largely from sample T6, while cluster 4 comprised Dx and EOI cells predominantly from sample T9. The clusters were annotated based on expression of known cell-type markers and some of the blast signature derived markers (Fig. S5a, b). The presence of cells in EOI samples that are transcriptionally similar and cluster together with blast cells from matched Dx samples indicate that these cells survive therapy and are residual blasts. Indeed, these two EOI samples were scored as MRD +  (Table S1). The blasts for the third MRD +  sample, T8, were not enriched in the matched Dx clusters (5 and 16) but largely segregated in cluster 14 which comprised blasts from other Dx and EOI samples.

The residual blast transcriptome is expected to reflect adjustment of T-ALL cells to induction therapy and consequent post-treatment persistence. We analyzed the pathways and regulators associated with genes that are differentially expressed in Dx and EOI blast cells from two MRD + samples (T6 and T9) (Fig. 3d). The PD-1 pathway was upregulated in EOI residual blast cells from both patients, indicative of T cell-exhaustion, possibly related to chemotherapy exposure^34,35^ (Fig. 3d). The Rho GDP dissociation inhibitor (RhoGDI) signaling pathway was upregulated while Cdc42 signaling was downregulated in residual blasts. Other downregulated pathways in residual blasts include Integrin signaling, IL-8 signaling and phospholipase signaling relative to Dx blast cells (Fig. 3d). Analysis of altered regulators revealed upregulation of the RAS family protooncogenes *HRAS* and *NRAS* in residual blasts (clusters 0 and 4) at EOI (Fig. 3e). The microRNAs miR 34a-5p and miR-182-5p, were also increased in residual blasts. Other regulators that are increased in residual blasts include the histone demethylase KDM5A, which can mediate chemoresistance and is being explored as a therapeutic target in AML^36,37^, the mitochondrial serine protease Caseinolytic Mitochondrial Matrix Peptidase Proteolytic Subunit (CLPP) and Immunity related GTPase M (IRGM); involved in autophagy^38^. We also generated a map of the interactants derived from the regulators analysis (Fig. 3f**)**. Genes such as *SOX4* and *VIM*, which are part of the Dx blast signature, are downstream of transcriptional regulators such as *HRAS* that were decreased in EOI blasts (Fig. 3f). Together, these modulated pathways and regulators suggest mechanisms of blast persistence after T-ALL induction therapy.

### Composition and transcriptome of BME differs significantly before and after induction therapy

**Figure 4:**
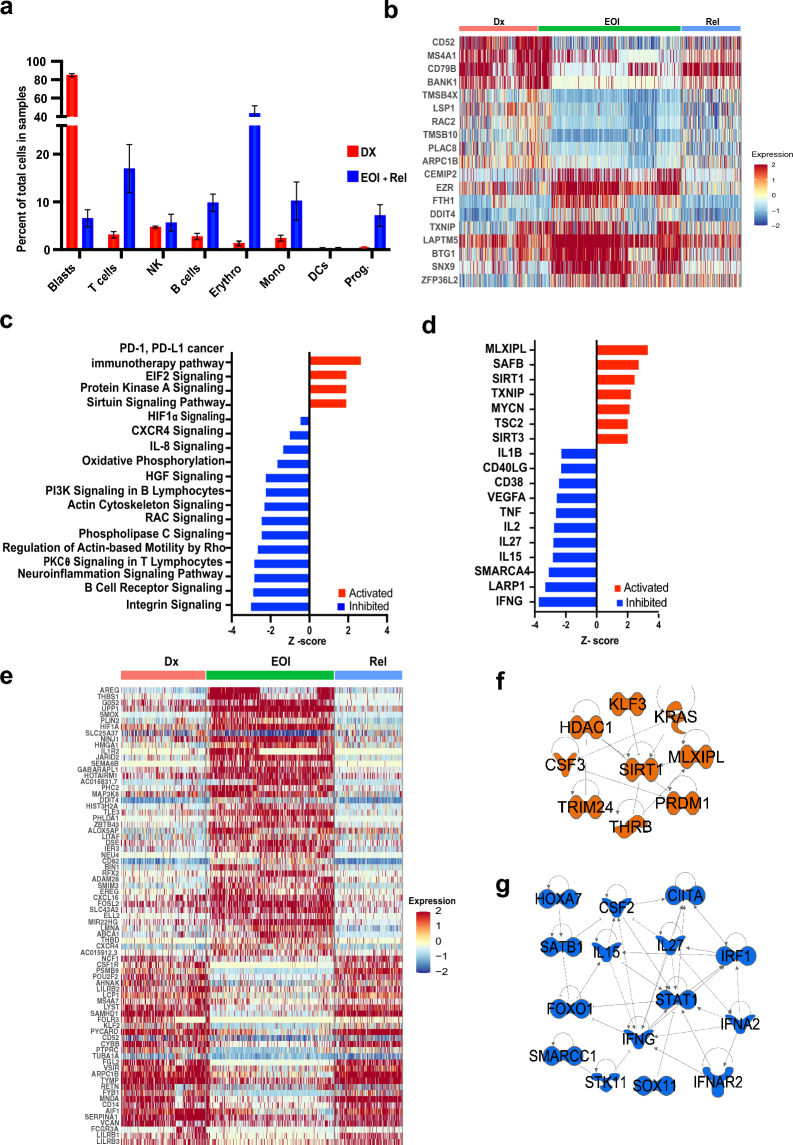
Comparing T-ALL BM microenvironment at Dx and EOI. (**a**) Bar-plot showing relative percent of blasts, T cells, NK cells, B cells, Erythroid cells, Monocytes, Dendritic cells (DCs) and Progenitor cells (Prog.) at Dx (red) and EOI + Relapse (blue). (**b**, **e**) Heatmaps showing scaled expression of genes derived from differential gene expression analysis between matched Dx and EOI (**b**) B cells from cluster 7 or (**e**) monocytes from clusters 13 and 17. Although the Relapse sample was not included in the analyses, it is shown in the heat map for comparison. Red, green and blue bars on top denote Dx, EOI and Relapse cells, respectively. (**c**) and (**d**) Bar plots showing Z-scores of selected pathways and upstream regulators that are activated (red) or inhibited (blue) in B cells at EOI relative to Dx based on differential gene expression generated using the IPA tool. Z-scores of selected regulators that are upregulated (**f**) or downregulated (**g**) in EOI monocytes relative to matched Dx samples.

Comparative analysis of paired Dx and EOI or relapse samples (T6-10, T11) also revealed differences in BM cellular composition (Fig. 4a). The clusters were annotated on the basis of expression of canonical markers for cell types and blast signature associated markers in the *CD3D* +  clusters (Fig. S5). As discussed in the previous section, blast cells comprised the majority of the samples at diagnosis and were drastically diminished post-treatment. In contrast, immune cells such as B cells, T cells, monocytes, and erythroid cells were more prominent in EOI samples (Fig. 4a). The erythroid cell clusters show clonal expansion at EOI, likely resulting from the elimination of blast cells (Fig. S6).

The T cell cluster (cluster 2; Fig. 3a) includes cells from all paired T6-T10 samples, and the relapse sample (Fig. S4) but had greater proportion of cells from the EOI and relapse samples (Fig. 3b). One MRD− sample (T7) had a ten-fold increase in the fraction of T cells and another MRD +  sample (T8) had an even higher expansion post treatment (Fig. S7a). The relapse sample (T11) also had a significantly higher fraction of T cells (Fig. S7a). Differential expression analysis between the EOI and Dx samples (after excluding relapse sample) in this cluster revealed genes *MT2A, DDIT4* that are associated with DNA damage and stress response*, BTG1,* the BTG anti proliferation factor 1 among those upregulated in T cells at EOI compared to diagnosis (Fig. S7b). *IL32*, *CRIP1, PFN1, S100A4,* and *ANXA1* were among those downregulated in EOI T cells.

B cells also exhibited changes in phenotype in EOI samples compared to matched Dx samples. Differential gene expression analysis was carried out between the EOI and Dx cells (excluding relapse sample cells) from the B cells cluster 7 (Fig. 3a) and the differentially expressing genes were plotted on a heatmap (Fig. 4b). Elevated expression of genes like *EZR, TXNIP, LAPTM5* was observed at EOI and while the levels of *MS4A1, CD52, CD79B, BANK1* were higher at Dx. BANK1 levels have been inversely correlated with B cell responses and antigen presentation^39^ and increased BANK1 may contribute to an immunosuppressive leukemia microenvironment at Dx. The lysosomal-associated protein transmembrane 5 (LAPTM5) negatively regulates B cell receptor expression and B cell activation^40^ and thioredoxin interacting protein (TXNIP) inhibits glucose uptake^41^ and expansion of germinal center B cells^42^. The B cell Translocation Gene1 (*BTG1*), which is involved in cell cycle arrest and adaptation to stress response, is also upregulated at EOI^43^. B cells from the relapse sample (T11) had a gene expression pattern similar to Dx samples and divergent from the EOI samples (Fig. 4b). Pathways analysis on the differentially expressed genes derived from the EOI vs Dx B cells (Fig. 4C) demonstrated upregulation of PD-1 and EIF2 signaling in EOI B cells, indicating therapy induced stress and induction of survival pathways (Fig. 4c). The Protein Kinase A and Sirtuin signaling pathways were also activated, suggesting modulation of metabolic and energy balance requirements. B cells also had downregulated HIF1α, oxidative phosphorylation, and PI3kinase signaling at EOI relative to Dx. Upstream regulators such as IFNG, TNF, VEGFA, IL1B and others which are consistent with an inflammatory phenotype are also downregulated at EOI relative to Dx (Fig. 4d). The carbohydrate response element binding protein MLXIPL is a transcription factor involved in glycolysis and lipogenesis in response to glucose, promotes B cell proliferation and is one of the top upregulated regulators in EOI B cells^44^. MLXIPL also induces TXNIP. SAFB, SIRT1, and SIRT3 were also increased in EOI B cells.

The monocyte/macrophage cells grouped into 2 separate clusters, 13 and 17 (Fig. 3a), both of which are positive for expression of *CD68, LYZ* and *CST3* as shown in Fig. S5a*.* Interestingly, cluster 13 is enriched with post treatment cells while cluster 17 is enriched with cells from Dx samples with the cells from relapse sample (T11) segregating along with the Dx cells in this cluster (Fig. S4). Figure 4e shows a heatmap of significant differentially expressed genes between EOI and Dx monocyte/macrophage cells. The relapse sample expression is also shown in the heatmap although it was not included for the evaluation of the differentially expressing genes. The relapse sample profiles are very similar to the Dx confirming the clustering behavior. The Dx group of cells are broadly *CD14*^+^ or *FCGR3A*^+^ *(CD16)*, while post therapy their expression is diminished. *CSF1R, NCF1, MS4A7, SAMHD1, ARPC1B*, are among the genes upregulated at Dx. Genes from the immune inhibitory LILRB family (*LILRB1-3*) are upregulated as is Versican (*VCAN*), which codes for epithelial to mesenchymal transition supporting proteoglycan, indicating that these cells fit the profile of tumor associated macrophages^45,46^. Post induction therapy, the EGFR ligand amphiregulin (*AREG*), uridine phophorylase, *UPP1*, thrombospondin1, G0/G1 switch2 (*G0S2*) are among the top enriched genes. AREG reduces phagocytosis-induced cell death in monocytes^47^. Long non-coding RNA HOTAIRM1 is increased and can modulate monocyte differentiation^48^. *THBS1*, which can suppress IL1β mRNA induction in macrophages^49^ and the gene for decoy receptor, *IL1R2*, a non-signaling receptor for IL1, are also upregulated in EOI cells. We also assessed M1/M2 lineage markers, but no prominent differences were observed, although *IL1B *was relatively higher at Dx (Fig. S8). The major regulators that are upregulated or downregulated in EOI monocytes are summarized in Fig. 4f,g respectively. Interestingly, there are parallels with the pattern observed in B cells as some of the regulators are seen to be similarly affected (upregulated/downregulated) at EOI relative to Dx. Therefore, different cell types in the immune microenvironment have some degree of overlap in their signaling environment.

### Cell signaling analysis reveals MRD association

**Figure 5:**
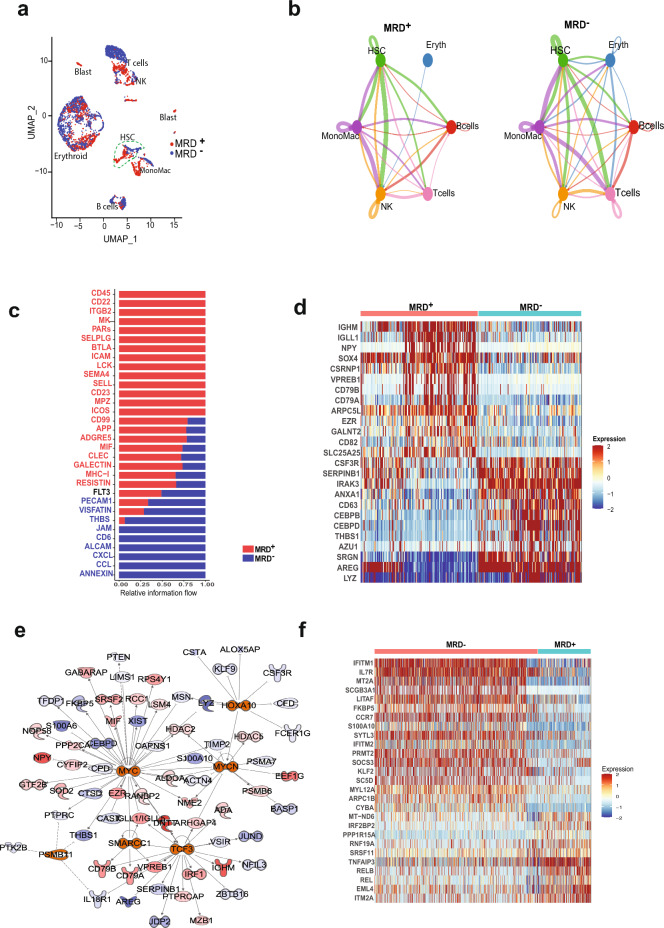
Analysis of cell–cell communication in EOI samples using Cell-Chat tool. (**a**) UMAP plot of the EOI samples colored by MRD status as shown in legend. Cell types of clusters are as labeled (**b**) Plot of cellular communication based on number of ligand-receptor interactions between different cell types. The width of the edge is proportional to number of interactions. The interactions in MRD +  and MRD− subsets in different cell types are shown side by side. (**c**) Bar plot showing the relative information flow within inferred networks and ranked based on the differences of overall information flow within these networks between MRD +  and MRD− groups. Red and blue denote pathways enriched in MRD +  and MRD− groups, respectively. (**d**) Heatmap showing differentially expressed genes between MRD +  vs MRD− cells in the HSC cluster. (**e**) Map of upstream regulators and their interactants derived from differentially expressed genes in MRD + vs MRD− cells in the HSC cluster determined using the IPA tool. Red denotes upregulation in MRD +  cells and blue denotes downregulation. (**f**) Heatmap showing differentially expressing genes between MRD− and MRD +  T cells.

We analyzed differences in cell signaling between cells in the immune microenvironment in the EOI MRD +  and MRD− groups. The EOI samples (T6–T10) were clustered together with assignment into MRD + (T6, T8, T9) and MRD− subgroups (T7, T10) Fig. 5a shows a UMAP of the EOI samples colored based on MRD status and distinct cluster types are identified based on the expression of known markers shown in Fig. S9. Cellular communication analysis was limited to cell types that were found in both the MRD +  and MRD− groups after removing patient specific, and blast cell clusters. Based on receptor and ligand expression between different cell types, a *CD34*^+^ cluster of hematopoietic stem cells (HSCs) demonstrated differential interactions with other cell populations in MRD +  and MRD− subsets. This HSC cluster showed overall heightened signaling interactions with other cell types, especially T cells, in MRD− samples relative to the MRD +  subset (Fig. 5b). Analysis of the interactions showed relative increase in THBS, JAM, CD6, Annexin and chemokine signaling post therapy in MRD− samples while MRD + samples showed higher CD45, CD22, ITGB2, and MK signaling, among others (Fig. 5c). Differential expression analysis in the HSC cluster identified higher expression of myeloid associated genes such as *CEBPB, CEBPD, AZU1* in the MRD− group relative to the MRD +  cells, which had higher expression of B-cell related genes such as *IGHM, VPREB1, CD79A/*B (Fig. 5d). Analysis of upstream regulators based on the differential gene signature between the MRD + and MRD− HSCs, demonstrated upregulation of *MYC* and *TCF3* activity and inhibition of *THBS1, CSF3* and *CEBPD* in MRD + samples compared to MRD− samples (Fig. 5e). Differential expression analysis of the T cells identified elevated expression of T naïve and memory markers like *IL7R*, *CCR7* in the MRD− subset (Fig. 5f). These results reveal marked differences in communication between different cell types in the EOI MRD + and MRD− BM as well as presence of MRD status specific immune cell types.

## Discussion

While bulk sequencing and mutational analysis have provided meaningful insights into T-ALL biology, detailed single cell characterization of leukemic blast populations and the immune microenvironment will help expand our understanding of T-ALL pathogenesis. To date, the T-ALL landscape has not been comprehensively investigated by single cell transcriptomics. In this study, we profiled Dx, EOI and relapse BM samples from pediatric T-ALL patients using scRNA-Seq analysis to gain insights into the expression profiles of and interactions between different BM cell types. ScRNA-Seq analyses enabled blast-specific transcriptome profiling as well as in-depth characterization of the leukemia microenvironment in pediatric T-ALL before and after induction therapy. Our approach demonstrates the feasibility and utility of single cell profiling of BME to dissect the T-ALL transcriptional landscape. With the immune microenvironment playing a significant role in the onset and clearance of malignancies, including leukemia, it is essential to study complete ecosystems of cancers using comprehensive single cell transcriptome, epigenetics, and proteome profiling approaches.

We observed patient-specific heterogeneity in leukemia cells present at Dx with scRNA-Seq data demonstrating patient-specific heterogeneity of blast clusters with variations in gene expression in different patients. Expression of distinct TCR isoforms in blasts from different patients contributes to patient-specific heterogeneity. Supervised analysis on the blast cell clusters depicted cluster-specific expression of multiple gene families- including *IFITM, CD1, NOTCH1*, topoisomerase, and oncogenes *JUN* and *FOS*- that might be contributing to inter-patient blast cell heterogeneity as well as differential response to treatment. Heterogeneity in maturation stages of leukemic clones is an established phenomena in T-ALL^50^. There was some degree of overlap in expression patterns between heterogenous blast clusters, allowing identification of commonly dysregulated networks and pathways in samples from different patients. Comparative analysis of transcriptome and flow cytometry data for known blast markers depicted concordance, providing secondary validation for our scRNA-Seq results. These results are encouraging and provide additional markers to explore for improved characterization/detection of T-ALL blasts. The differences observed where low levels of expression are reported in flow data and scRNA-Seq assay, could in part arise from the fact that immunophenotyping data reflects qualitative assessment of protein expression subject to differences in interpretation and also possibly, due to protein and mRNA levels not necessarily correlating.

Comparative analysis of Dx blast cells with healthy BM cells identified a set of overexpressed genes in the heterogenous T-ALL blasts. These included genes that impact microtubule dynamics and cell cycle progression (*STMN1*)^51^, cancer stemness (*SOX4*)^52^, cell development and proliferation (*HES4*)^53^, cell adhesion (*CD99*)^54^, and a novel gene with unknown function (*ARMH1/C1orf228*). Overexpression of some of the blast signature genes was significantly associated with poorer event free survival in patients with T-ALL, consistent with modulation of key pathways related to tumor cell growth, proliferation, and migration. The association of SOX4 as a central mediator of tumor cell survival and chemoresistance by activation of PI3K/AKT and MAPK signaling has been extensively evaluated in B-cell acute lymphoblastic and myeloid leukemias^55,56^ but this paradigm has not been explored in T-ALL. Oxidative phosphorylation pathway, significantly upregulated in most blast cell clusters, has also previously been shown to be upregulated in certain breast cancers^57^ and Hodgkin lymphoma^58^. In AML, the suppression of oxidative phosphorylation has been shown to target leukemia stem cells thereby enhancing remission^59^. Other commonly upregulated pathways like NFAT, EIF2, CXCR4 signaling have also been implicated in T-ALL^60,61^. Further exploration and validation of the blast signature genes and pathways could identify novel diagnostic and therapeutic targets for improved outcomes in T-ALL.

Importantly, comparative analysis of scRNA-seq data from Dx and EOI blast clusters enabled identification and characterization of therapy resistant residual blast cells at EOI. This is significant because MRD assessment has emerged as a powerful approach to predict long-term outcome in pediatric cancer patients. T-ALL patients with MRD at EOI have significantly inferior outcomes^62^. Multiple retrospective studies have demonstrated post-induction MRD status as a reliable predictor of relapse^63–65^. Our analysis identified genes, pathways and processes that are enhanced in residual blasts present at EOI, including upregulation of PD-1 and RHO-GDI signaling, and shed light on genes, pathways and processes potentially mediating resistance to therapy. The Rho GDP dissociation inhibitor (RhoGDI) signaling pathway has been implicated in resistance of tumor cells to chemotherapy-induced apoptosis^66^. RhoGDIs bind and sequester RhoGTPases such as Cdc42 in their inactive GDP-bound form^67^ and Cdc42 signaling was downregulated in residual blasts. Cdc42 has been associated with reduced proliferation and apoptotic death in normal T cells^68^. It will be interesting to explore whether some of the regulators, such as miR34a-5p, upregulated in residual blasts, have favorable prognosis as higher expression has been associated with better overall survival in some cancers, such as colon cancer^69^. MiR-182-5p, also upregulated in the residual blasts, is associated with poorer prognosis with increased expression in breast and prostate cancer, whereas, in other cancers, such as gastric cancer, downregulation is associated with poorer prognosis^70–72^. Analysis of dysregulated genes and pathways in residual cells could also be useful to evaluate disease burden as an indicator of clinical response.

Analysis of non-cancerous cells, including immune populations, revealed that cells of erythroid, lymphoid, and myeloid lineages were significantly expanded at EOI, coincident with decreases in leukemic blasts. Transcriptional profiles in monocytes/macrophages, B cells and T cells were also divergent in Dx and EOI samples. Immune cell profile is a key determinant of therapeutic response and can enable tumor cells to escape surveillance^73,74^. Leukemia cells can modulate their microenvironment towards immune suppressive phenotypes and thereby contribute to resistance mechanisms^34,75^. Understanding drivers that modulate the leukemia microenvironment to suppress anti-leukemia immunity is desirable to prevent relapse and improve outcomes. Comparative analysis of cells fromDx and EOI samples yielded some interesting molecular insights suggesting re-establishment of a functional immune microenvironment that favors clearance of leukemia cells. Upregulation of genes such as the tumor suppressor *TXNIP* and *BTG1* involved in cell cycle arrest and adaptation to stress response and downregulation of *BANK1,* the levels of which are inversely correlated with B cell responses and antigen presentation^39^ in EOI B cells imply overcoming the immune suppressive environment to favor clearance of leukemia cells. Downregulation of HIF1α, oxidative phosphorylation, and PI3kinase pathways at EOI along with the regulators IFNG, TNF, VEGFA, IL1B in B cells is likely to impede leukemia supportive pro-inflammatory signaling. Monocytes also showed downregulated genes from the immune inhibitory LILRB family and the epithelial to mesenchymal transition supporting proteoglycan, Versican at EOI relative to Dx. A subset of the upstream regulatory molecules that were increased in B cells such as MLXIPL, SIRT1 were also upregulated in EOI monocytes while IFNG, IL27 were downregulated. Thus, at least some of the changes in the immune microenvironment after treatment are consistent in different cell types.

Both B cell and monocyte populations in the relapse sample had a significantly divergent profile compared to EOI samples. Interestingly, the B cells and monocytes from the relapse sample showed much less variation in their transcriptome profiles compared to Dx samples. While this analysis was limited to a single sample, behavior of cells in the relapse BM sample appears to reflect the behavior of cells supporting a leukemia rich environment, as in the Dx samples, despite the absence of an overwhelming blast cell population in this particular sample (T11). In contrast, MRD +  and MRD− EOI samples clustered together, despite the presence of blast cells in MRD +  samples. In this context, upregulation of thrombospondin (THBS) signaling in the MRD negative subset is interesting since it promotes differentiation and decreases proliferation in a human myeloid leukemia cell line and peptides derived from the C terminus of this protein also contribute to immunogenic cell death^76,77^. The presence of excessive blast cells in leukemic bone marrows is very likely to disrupt steady state cell-to-cell communications on account of diminishing healthy immune populations. Despite the overall similarity between MRD +  and MRD− samples, a more granular analysis of cellular communication and interactions based on expression of ligands and receptors demonstrated differences in signaling patterns between MRD +  and MRD− samples, which could be influenced by the presence of residual blast cells. In particular, HSCs in MRD +  marrow expressed more B cell related genes, while HSCs in MRD− marrow expressed more myeloid genes, consistent with marrow reconstitution versus an ongoing disease state. Based on these preliminary insights, we postulate that evaluation of immune cell signatures after induction therapy can offer clues into therapy associated changes and potentially determining the presence of residual disease. Such studies are likely to yield new targets with potential to improve long term outcomes in T-ALL. Given the small number of samples analyzed in the present study, we plan to further validate the identified genes, pathways and regulatory networks in larger pediatric T-ALL datasets by incorporating single cell multiomic analysis.

## Conclusions

Using scRNA-Seq data analysis we have derived a pediatric T-ALL blast-associated gene signature and examined patient specific heterogeneity of blast cells in pediatric T-ALL bone marrow at diagnosis. We characterized residual blast cells at EOI and performed analysis of the transcriptome, pathways, and regulators involved in specific immune cell types at Dx, EOI and relapse. Analysis of cell-to-cell communication networks provided interesting insights into altered signaling in the context of overt leukemia and minimal residual disease. These results provide critical first insights into the pediatric T-ALL transcriptional landscape at single cell resolution and identify potential therapeutic targets to improve outcomes for children with T-ALL.

## Methods

### Clinical samples

Viably frozen de-identified BM samples and associated clinical data (Table S1) from 11 pediatric patients (1.7–16.5 years old) with T-ALL were obtained from the Aflac Leukemia and Lymphoma Biorepository at Children’s Healthcare of Atlanta (CHOA), GA. Studies using human samples were conducted in accordance with relevant guidelines and regulations. Experimental protocols were approved by the Emory University Institutional Review Board (Protocol #00,034,535). All samples were collected after written informed consent/assent was obtained from patients and/or their legal guardian(s). Ten patients maintained a CR and are still alive (T1-T10). One patient (T11) relapsed twice and did not survive. A total of 16 samples were collected from the 11 patients: 10 at diagnosis (Dx), 5 after therapy (EOI) and 1 at relapse. Three EOI samples were collected from MRD +  patients (T6, T8, T9) and two were MRD− (T7, T10). A publicly available scRNA-Seq healthy BM dataset was used as control for comparative analysis (https://www.ncbi.nlm.nih.gov/geo/query/acc.cgi?acc=GSE154109) with T-ALL samples^24^.

### Single cell RNA sequencing

BM single cells were captured together with uniquely barcoded primer beads in droplets in the Chromium Controller (10 ×  Genomics, 110,211). The scRNA-Seq libraries were prepared using the 10x Genomics Chromium single cell 3’v3 and 5’v1 reagent kits (10 ×  Genomics, 1,000,075 and 1,000,006) and sequencing was performed using massively parallel sequencing on the Novaseq S4 platform producing > 20,000 reads per cell.

### Single cell RNA sequencing data processing

Raw scRNA-Seq data was demultiplexed, aligned to the reference human genome (GRCh38) and processed for single cell gene counting using the Cell Ranger Software from 10x  Genomics Inc. The single cell count data was normalized using SC Transform function in Seurat v3.0 Bioconductor package^78^. The quality filtering on scRNA-Seq data was performed by multiple parameters filtering out low quality cells including: > 25% of mitochondrial genes, cells expressing low number of genes (< 200 genes) and genes only uniquely expressed in < 5 cells. In order to determine overall relationship among the cells, the unsupervised analysis using principal component analysis (PCA) was performed on variable genes to identify principal components with significant variation that were used as input for clustering and Uniform Manifold Approximation and Projection (UMAP) analysis^79^.

### Cell type characterization and analysis

Cells with similar transcriptome profiles were clustered together, and based on the expression of known lineage markers, the clusters were subsequently annotated as T cells (*CD3D*), B cells (*CD19, CD79A, MS4A1*), monocytes and macrophages (*CD14, CD68*) and erythroid cells *(HBB, HBD*). Putative leukemic blasts were annotated based on known T-ALL blast marker genes such as *CD99, CD5, CD3D,* and *CD7*. The analysis of single cell landscape of T-ALL blast and non-blast cells was performed using the Seurat R package^78^. In an unbiased approach, cell types and subtypes were characterized by generating the differentially expressed genes with ‘FindMarkers’ function in Seurat using non-parametric Wilcox rank test (*P* value < 0.01) and fold change > 0.25. The cluster distinguishing markers, dot plots, feature plots, violin plots and heatmaps were generated using the Seurat inbuilt ‘*FindAllMarkers*’, ‘*DotPlot*’, ‘*FeaturePlot*’, ‘*VlnPlot*’ and ‘*DoHeatmap*’ functions respectively. Heatmaps with downsampling were generated by including the ‘*downsample*’ argument in *DoHeatmap* function to randomly downsample to a set number of cells (as indicated in the figure legends) from respective clusters.

### Generation of T-ALL blast associated gene signature

T-ALL blast clusters were identified based on expression of known T-ALL blast markers, overexpression of TCR isoforms and patient specific heterogeneity as described in the results section. The Dx samples were clustered with healthy control BM samples from a recent study^24^ to identify key genes associated with T-ALL blasts. Using the ‘FindMarkers’ function in Seurat, we generated a blast associated expression signature by comparing the gene expression profiles of blast associated clusters with healthy BM T cell clusters.

### Pathways and systems biology analysis

To further characterize the T-ALL BME and understand the molecular mechanism of disease progression, we performed pathways and systems biology analysis on the transcripts that are significantly overexpressed in the blast cells as well as in the immune cells of the T-ALL BME. Pathways and systems biology analysis was performed using the Ingenuity Pathway Analysis software package (IPA 9.0) (Qiagen). A detailed description of IPA analysis is available at the Ingenuity Systems’ website (http//www.ingenuity.com). Systems biology analysis was performed using upstream regulators enrichment approach to identify upstream transcriptional regulators that can explain observed transcriptome changes. Regulatory analysis helps in identifying significantly activated or inhibited transcriptional regulators based on upregulation or downregulation of their target genes. The significance of transcriptional regulator activation/inhibition was determined using one-tailed Fisher’s Exact test. Regulators with a *P* value < 0.01 and absolute Z-score ≥ 1 were considered statistically significant.

### Analysis of cell–cell communication

The CellChat R tool (v 1.0.0, http://www.cellchat.org/) was used to infer cell–cell communication within the EOI MRD + and MRD− subsets and to compare their communication networks^80^. This tool uses a ligand-receptor interaction database to infer communications between different cell types in a dataset. It identifies the pathways showing significantly altered signaling between different cell types in the dataset, as well as the strength and number of communications it estimates between cell types.

### Survival analysis using select T-ALL blast associated genes

To determine the association of blast genes with clinical outcomes in T-ALL, we performed survival analyses using the recently published Survival Genie tool^32^. The TARGET ALL Phase 2 dataset from GDC data portal [https://portal.gdc.cancer.gov/] was selected for analysis. Primary pediatric BM or peripheral blood mononuclear cells (PBMCs) collected at diagnosis with available clinical and normalized FPKM expression counts were obtained. We examined the association of both the combined (top25) and individual blast genes on clinical outcome in the primary cohort. For gene-level analysis, an optimal cut point (cutp) was estimated based on martingale residuals^81^ using the ‘survMisc’ package to separate the patients into high and low gene expression groups. For combined analysis, we used the non-parametric method, single sample gene set enrichment analysis (ssGSEA) as implemented in the GSVA package^82^ to derive gene-set activity score. Tumor samples were categorized into high and low activity groups using either the median or an optimal cut point of the ssGSEA score as cut-off. Statistical analysis of event-free survival (EFS) was performed using the ‘survival’ and ‘survminer’ packages from R/Bioconductor. Kaplan Meier Survival curves were used to estimate EFS using survfit function and a log-rank test was done to compute differences between the high and low groups. Cox proportional hazards regression was performed on the data set using coxph function. Hazard ratios (HRs) were calculated with low expression as reference for gene-based survival associations, and MRD negative or CNS1 (CNS Negative) as references for clinical-based survival associations. The results were considered significant if the *P* values from log rank and Wald test were below 0.05.

## Supplementary Information

Supplementary Information.

## Data Availability

The datasets generated and analyzed in this study have been deposited in NCBI’s Gene Expression Omnibus under accession code GSE227122.
