# Supplementary material for: Pediatric T-cell acute lymphoblastic leukemia blast signature and MRD associated immune environment changes defined by single cell transcriptomics analysis
Source: Sci Rep. 2023 Aug 2;13:12556. doi: 10.1038/s41598-023-39152-z (PMC10397284; doi:10.1038/s41598-023-39152-z)
Supplement: Supplementary file 1 — Supplementary Information. [file 41598_2023_39152_MOESM1_ESM.pdf]

Supplementary File

Pediatric T-cell acute lymphoblastic leukemia blast signature and MRD associated immune environment changes defined by single cell transcriptomics analysis.

Swati S. Bhasin[1,2]*, Beena E. Thomas[1,2], Ryan J. Summers[1,2], Debasree Sarkar[1,3], Hope Mumme[1,3], William Pilcher [4], Mohamed Emam[2], Sunil S. Raikar[1,2], Sunita I. Park[5], Sharon M. Castellino[1,2], Douglas K. Graham[1,2], Manoj K. Bhasin[1-4], Deborah DeRyckere[1,2].

***Corresponding author**

1. Aflac Cancer and Blood Disorders Center, Children's Healthcare of Atlanta, Atlanta, GA
2. Department of Pediatrics, Emory University School of Medicine, Atlanta, GA
3. Department of Biomedical Informatics, Emory University School of Medicine, Atlanta, GA
4. Wallace H Coulter Department of Biomedical Engineering, Georgia Institute of Technology, Atlanta, GA
5. Department of Pathology, Children's Healthcare of Atlanta, Department of Pathology and Laboratory Medicine, Emory University School of Medicine, Atlanta, GA

Supplementary Figures

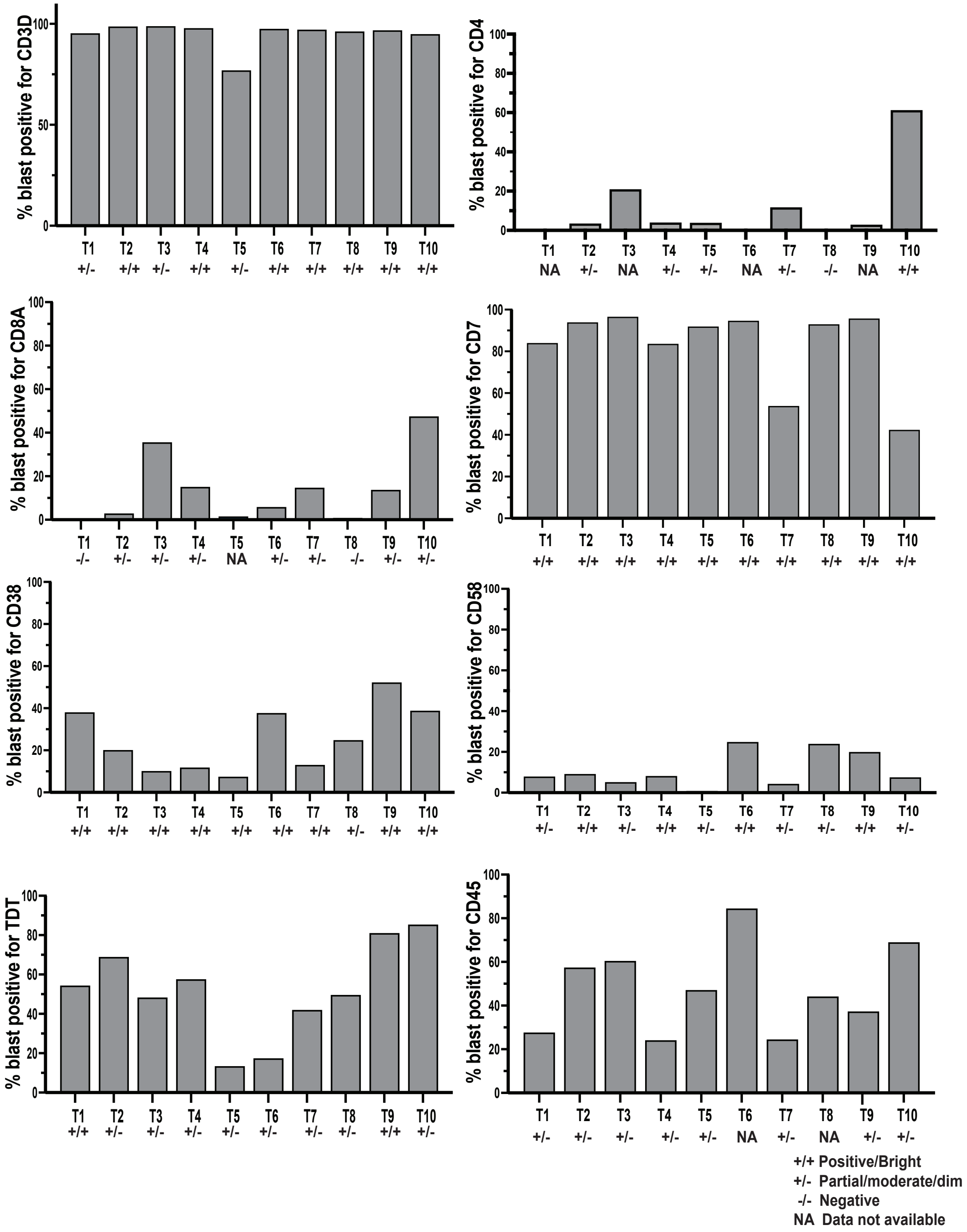

Figure S1 Bar plots showing percent of blast cells expressing a given marker (as labeled along y- axis) in each patient bone marrow sample (at diagnosis), as labeled along the x-axis. The labels +/+, +/-, -/-, and NA below sample identity along the x-axis, indicate surface protein flow cytometric assignments into positive/bright, partial/moderate/dim, negative and data not available respectively.

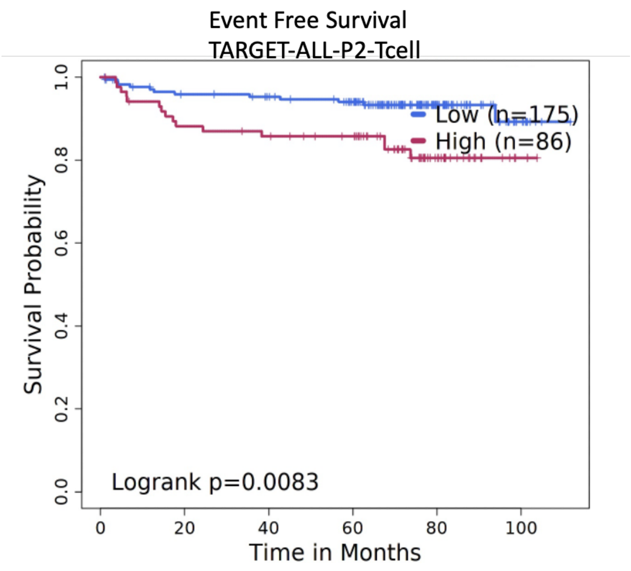

Figure S2. Survival analysis depicting combined over expression of the T-ALL blast expressing top 25 genes in the TARGET ALL phase2 cohort is significantly associated with poorer event free survival *(HR=2.7, LR P value=0.008).*

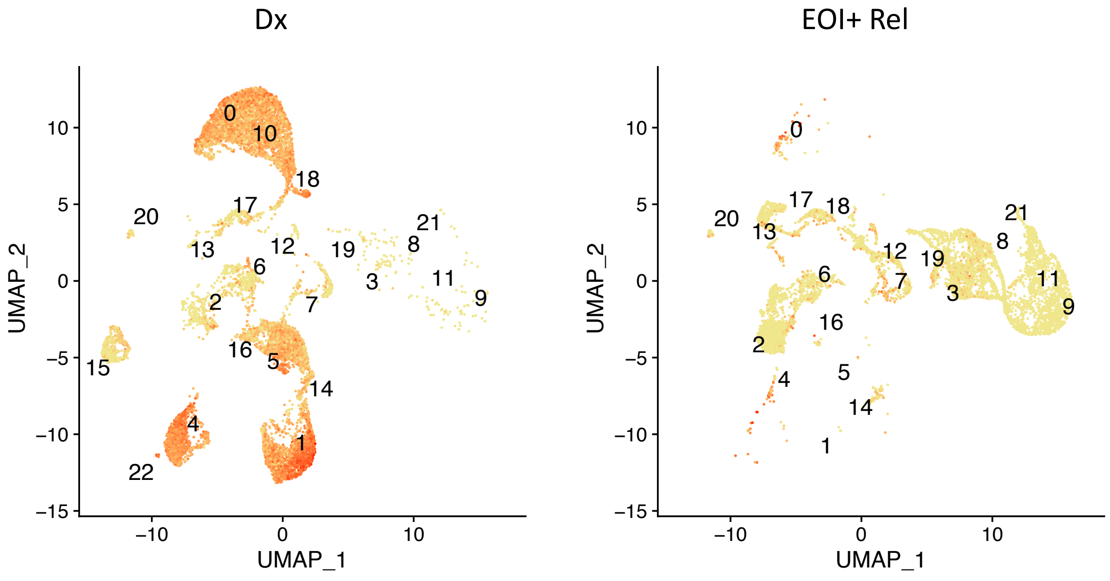

Figure S3. Split UMAP plot of the Dx, EOI, Relapse merged object showing Dx cells on the left and EOI, Relapse cells on the right. Plot shows combined expression (denoted by intense orange) of top genes derived from the blast signature in the Dx, EOI, Relapse UMAPs. Orange color indicates high expression and yellow color indicates low expression.

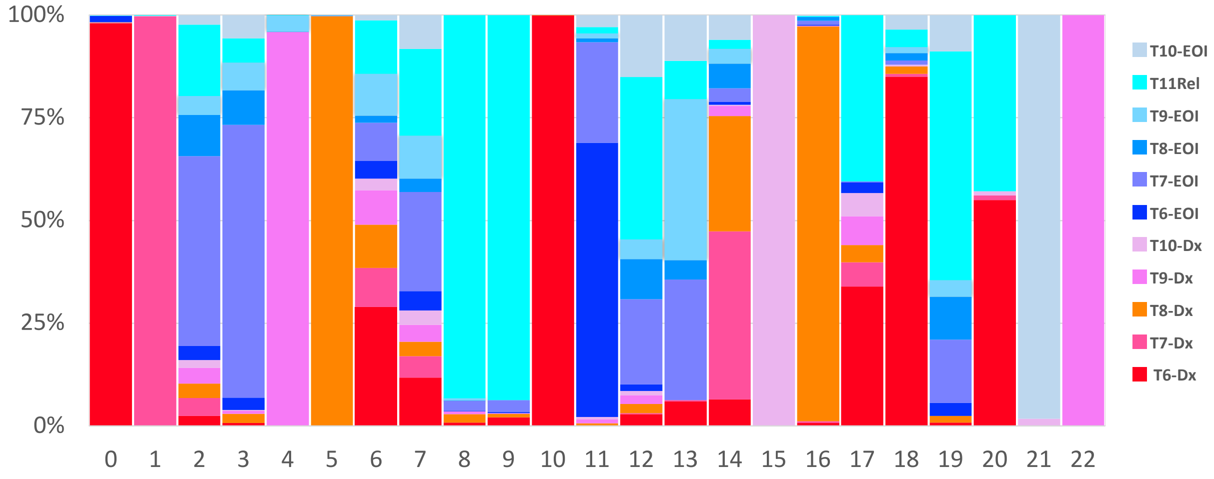

Figure S4. Bar-plot of cluster-wise distribution in the merged Dx, EOI, Relapse object of samples T6-T11 (Dx, matched EOI, and Relapse). x-axis denotes cluster number while y-axis represents percent composition by sample. Each sample is shown in a different color as noted in the legend on the right. Matched Dx and EOI samples have same number and with Dx or EOI suffix, respectively.

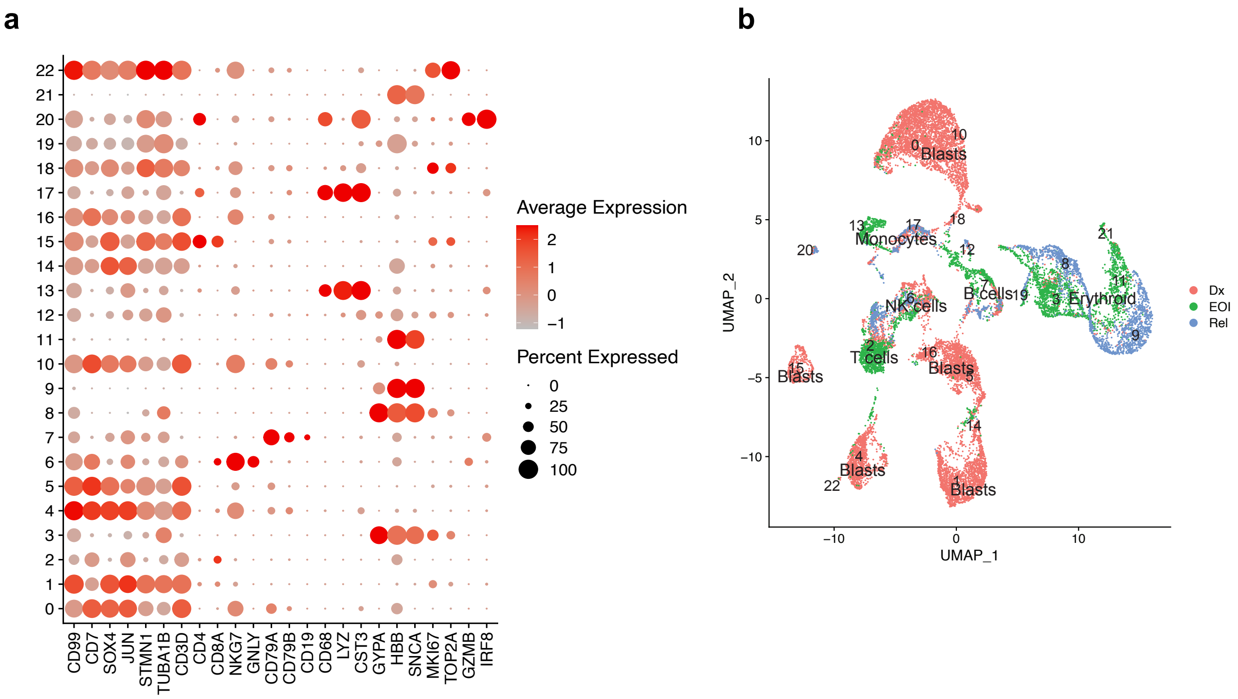

Figure S5. (a) Dot-Plot showing cluster-wise expression of markers used for annotation of Dx, EOI, Relapse merged object. **(b)** Merged Dx, EOI, Relapse UMAP plot showing Dx, EOI, and Relapse cells colored in red, green and blue colors, respectively. The cluster numbers are as shown, and major cell types are labeled based on the canonical marker expression in (**a**).

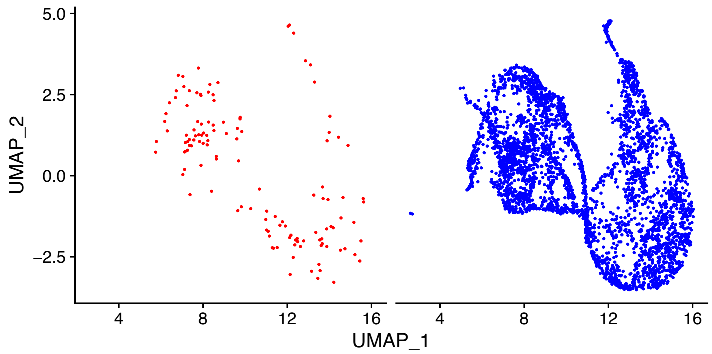

Figure S6. Split UMAP plot showing the distribution of the erythroid cell clusters. Dx cells are on the left (red) and EOI, Relapse cells (blue) are on the right.

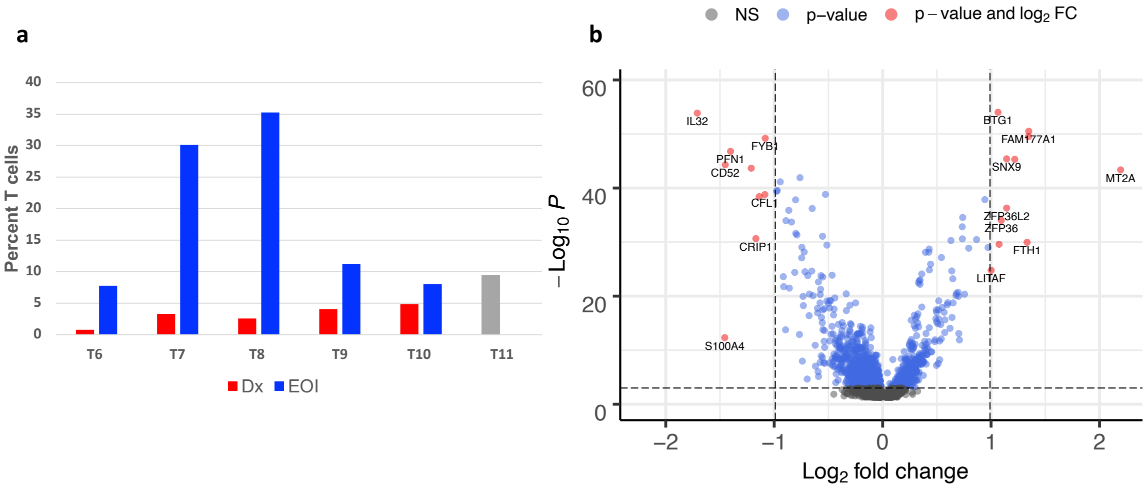

Figure S7. (**a**) Percent T cells of total cells captured by scRNAseq in each sample and (**b**)Volcano plot showing differentially expressed genes between T-cells before and after induction therapy generated using the 'EnhancedVolcano' package in R/Bioconductor. Significantly upregulated genes at EOI relative to Dx are shown on the right (positive fold change) while downregulated genes are shown on the left (negative fold change) with grey, blue and red color dots indicating genes that are not significant (NS), P value significant and P value significant with log2 fold change >1, respectively.

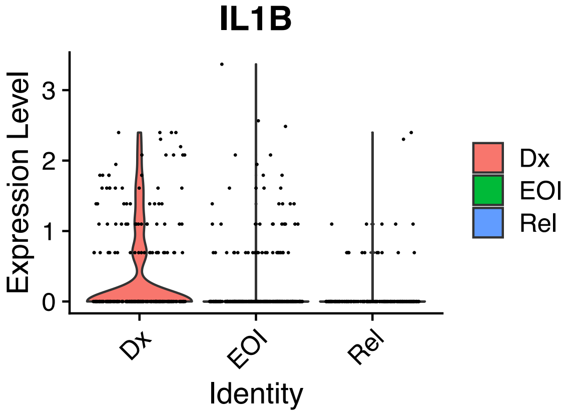

Figure S8. Violin Plot showing *IL1B* expression split across Dx, EOI and Relapse samples in Monocyte clusters 13 and 17 combined

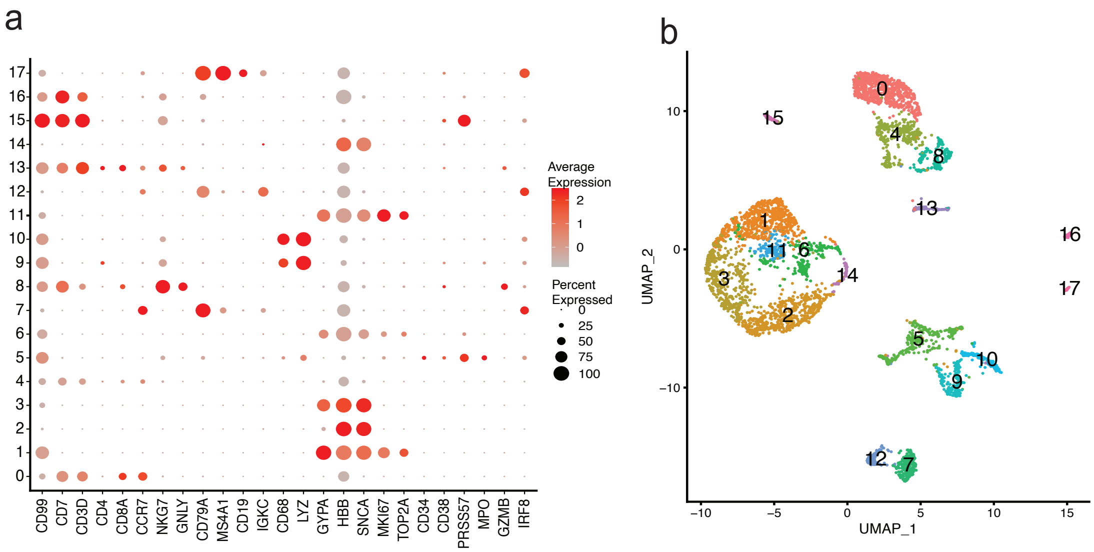

Figure S9 (a) Dot plot showing cluster-wise expression of markers used for annotation of the merged EOI time point samples, T6-T10. (**b**) Merged EOI UMAP plot showing clusters 0-17 in different colors.

.

Supplementary tables

Table S1

T-ALL patient information for samples T1-T11, included in this study. Samples were collected from patients T6-T10 at diagnosis (Dx) and end of induction (EOI). All other patients provided a single sample at Dx or relapse (rel).

Sample ID	Age at Dx (years)	Race	New Dx or Rel	Sample time point [Dx, EOI, or Rel]	Initial WBC at Dx ($x10^3/\mu l$)	PB Blasts at Dx (%)	BMA Blasts at Dx (%)	EOI MRD	EOI MRD %	Induction Regimen	Has patient relapsed?	Karyotype	ETP-ALL or near ETP-ALL features	CNS disease	CNS leukemia disease (at Dx)
T1	10	Black or African American	New Dx	Dx	140.02	80.3	97	Negative	0	AALL1231	No	46,XY[59]	Yes (one blast population is ETP ALL)	No	CNS 1
T2	16.5	Black or African American	New Dx	Dx	298.3	90.3	94	Negative	0	AALL1231	No	46, XY[10]	No	Yes	CNS 2
T3	1.7	Black or African American	New Dx	Dx	130.3	63.5	85.8	Negative	0	AALL1231	No	46, XX,t(11;14)(p13;q11.2)(10)/46,XX[23]	No	No	CNS 1
T4	3.2	Asian	New Dx	Dx	103.09	88.3	96.1	Negative	0	AALL1231	No	46,XX,i(9)(q10)[5]/46,XX[20]	No	Yes	CNS 2
T5	13.5	White	New Dx	Dx	100.8	83.7	81.5	Positive	0.07	AALL0434	No	46,XX,t(10;11)(p13;q21)[5]/46,XX[28]	No	No	CNS 1
T6	5	Black or African American	New Dx	Dx, EOI	101.4	8	72.9	Positive	5.4	AALL1231	No	46, XY[30]	No	Yes	CNS 2
T7	6.4	White	New Dx	Dx, EOI	127	81.3	95.2	Negative	0	AALL1231	No	46,XY,del(10)(q23)[2]/46,XY[18]	No	No	CNS 1
T8	8	White	New Dx	Dx, EOI	14.77	63	64.6	Positive	8	AALL0434	No	50~51,XY,+8,+10,+11,der(11)t(9;11)(q21;p12),+13,+19,+22[cp20]/46,XY[10]	Yes (near ETP ALL)	No	CNS 1
T9	7.7	Black or African American	New Dx*	Dx, EOI	113.1	89.7	75.6	Positive	5	Nel/VP/CTX	No	46,XY[20]	Yes (near ETP ALL like)	Yes	CNS 2
T10	4.1	White	New Dx	Dx, EOI	84.44	57.7	95.4	Negative	0	AALL0434	No	46,XX,30	No	Yes	CNS 2
T11	11.1	Black or African American	Rel	Rel	122.63	78.9	N/A	Negative	0	AALL1231	Yes	46,XY[20]	No	Yes	CNS 3

Dx - Diagnosis, Rel - Relapse, EOI - End of Induction, WBC - White Blood Cell, PB - Peripheral Blood, BMA - Bone Marrow Aspirate, MRD - Minimal Residual Disease, CNS - Central Nervous System, *Secondary Malignancy

Table S2

Table showing the top 25 cluster distinguishing genes for each of the 0-20 clusters of the merged TALL diagnosis samples and Controls object. The top row of each column indicates cluster number.

0	1	2	3	4	5	6	7	8	9	10
TRDV1	TRAF4	TRBV11-2	TRGV4	IFI27	TRGC2	RPS29	TRBV20-1	TRBV12-5	GNLY	S100A9
IL32	EIF1	MAL	TMEM131L	JUN	ZBTB16	RPL21	HES4	FXYD2	NKG7	S100A8
IFITM1	CHI3L2	HES4	ARMH1	SCGB3A1	MT-ATP8	RPL41	FOS	TRBV2	CCL5	LYZ
IFITM2	CHD1	PPIA	CDK6	PMAIP1	RPL22L1	RPS20	XG	S100A10	GZMB	CST3
TRGV5	HLA-B	FAM173A	MYO7B	CHI3L2	PDLIM1	RPS10	TUBB	MEST	CST7	AC020656.1
KRT1	SFPQ	CD1E	CLEC11A	GADD45B	ITM2A	RPL27A	MAL	CRIP1	PRF1	TYROBP
AHNAK	CLEC2B	CD1B	PGM2L1	CD99	STXBP2	RPL31	SERPINE1	PTCRA	FGFBP2	FTL
MYOM2	RNF11	GAPDH	TMSB10	H1FX	GNA15	TRAC	STMN1	ID3	CTSW	S100A6
LTB	SLC25A6	FABP5	EVL	ATF3	SCGB3A1	RPL38	GAPDH	GAPDH	KLRD1	FCN1
GZMA	RELB	ATP5MC3	PRMT7	FOS	TPM4	RPS27	H2AFZ	PTMS	KLRB1	S100A4
TRDC	LDLR	TRBC1	TFDP2	GAPDH	TRDC	RPL13A	TRBV6-6	H2AFZ	GZMH	CTSS
PTPRCAP	KDM5B	HMGA1	DNTT	TUBA1B	BLK	RPL36A	TXN	TYMS	GZMA	S100A12
KLRB1	MT-ND5	RPLP0	CD1B	IER2	PTTG1IP	RPS17	MZB1	HMGN2	TYROBP	FCER1G
CTSW	ZNF292	HSPD1	TRGC2	UBC	TRGV2	RPL7	CD99	HES4	CCL4	LGALS1
HLA-E	RTN4	ZFAS1	STT3B	ENO1	EEF2	RPLP2	HIST1H2BK	VIM	B2M	COTL1
CD69	RB1CC1	SIVA1	AC245060.5	GYPC	MT-ND2	RPL34	TYMS	BEX1	HLA-C	S100A11
S100A11	CCNI	RPS26	TSC22D3	GADD45A	HPGD	RPL23	CLIP3	TSC22D1	CMC1	HLA-DRA
ANXA1	CHD2	DUT	CD1E	MIF	RPS19	RPL9	CDT1	ID1	SRGN	HLA-DRB1
CRIP1	TSPYL2	NME2	PTP4A2	H3F3B	CIRBP	RPL23A	MYO7B	MIF	HLA-B	SRGN
SELL	EIF4G2	MZT2B	ARL4C	KLF6	MT-CYB	RPL27	BTF3	TXN	HOPX	VCAN
CD52	SLC2A3	RCBTB2	DDX17	PPP1R15A	EEF1G	RPS6	VAT1	TUBB	HLA-A	SERPINA1
ZFP36L2	SRSF11	HMGB2	TCF7	H2AFY	CD82	RPL37A	DHFR	TUBA1B	FCER1G	MNDA
LSP1	RBM38	H2AFZ	GALNT2	H1F0	RPL18	TOMM7	TUBA1B	HMGB2	ID2	CD74
SLFN5	RPS4X	TUBB	TASP1	TPI1	SELENOW	RPL39	HMGN2	Z93241.1	FCGR3A	LST1
CD3E	HSP90B1	TUBA1B	MBNL1	HMGN2	CALR	RPS25	DUSP1	HSPA5	SPON2	TYMP

11	12	13	14	15	16	17	18	19	20
IGLL1	CD74	IGHM	CCL5	AL365440.2	MT-ND4L	RPS27A	HBB	GZMK	GZMB
ACY3	IGHM	HLA-DRA	GZMK	CD1A	MT-CO2	HLA-B	HBD	LGALS3	JCHAIN
TRDV3	HLA-DRA	CD74	RPS29	TOP2A	MT-CO1	TOMM7	HBA1	CD8B	IRF8
TRGV3	CD79B	IGKC	TRAC	SMPD3	MT-CYB	RPS25	HBA2	CD27	PLD4
SOCS2	TCL1A	HLA-DPB1	RPL41	RTKN2	MT-CO3	RPL14	CA1	CST7	LILRA4
RGPD3	HLA-DRB1	IGLC2	RPL27A	AL138899.1	MT-ND1	HLA-C	HBM	CCL5	CCDC50
CAT	IGLC2	HLA-DPA1	B2M	CENPF	MT-ATP6	RPS14	AHSP	GZMA	TCF4
PRSS57	HLA-DPB1	HLA-DRB1	CXCR4	CD1E	MT-ND5	RPL11	ALAS2	ISG20	RNASE6
DUSP6	CD79A	CD79A	CST7	NUSAP1	MT-ATP8	RPL32	SLC25A37	COTL1	SPIB
DDIT4	HLA-DPA1	IGHD	RPS20	XIST	MT-ND6	RPL30	BLVRB	CNN2	APP
DNTT	VPREB3	HLA-DQB1	RPS27	TFDP2	MT-ND3	JUNB	PRDX2	CD81	UGCG
TUBA1A	HLA-DQB1	CD79B	RPL13A	MKI67	MT-ND2	RPL13	CA2	SRGN	SERPINF1
TNFAIP3	HLA-DQA1	MS4A1	GZMA	PCLAF	MT-ND4	SARAF	SLC4A1	CD74	HLA-DPA1
GNA15	MS4A1	RPS29	RPL21	TRBC2	XIST	KLF2	SLC25A39	S100A4	IRF7
TPM4	CD24	CXCR4	RPL31	NUCKS1	HES4	RPS12	SNCA	MT2A	CST3
SELENOW	IGKC	RPL41	RPL38	DEK	PGGHG	B2M	GYPA	HLA-A	GRN
MS4A4E	IGLC3	TCL1A	RPS10	NUCB2	SRSF10	RPS3A	HMBS	S100A6	FCER1G
TSC22D3	HLA-DMA	CD37	DUSP2	STMN1	SF1	LEPROTL1	BSG	HLA-C	HLA-DRB1
EXD3	LAPTM5	IGLC3	HLA-C	GNAS	HNRNPH1	IL7R	SELENBP1	ARPC1B	HLA-DRA
GSTP1	CD37	RPL13A	HLA-A	HIST1H4C	MZB1	MT-ND3	FECH	TMSB4X	HLA-DPB1
RGPD4	RGS2	RPS10	HLA-B	HMGB2	FUS	RPS4Y1	HEMGN	NKG7	NPC2
VPREB1	CD83	RPL21	RPL36A	MT-ATP6	CCNL1	RPS26	LGALS3	LIMD2	CD74
ENO1	IGLL1	RPS27	RPS17	MT-ND4	NKTR	DNAJB1	TRIM58	LGALS1	ALOX5AP
PTGER3	VPREB1	RPS20	CMC1	MT-ND3	MIR181A1HG	MT-ND4	GYPC	PTPRCAP	ITM2C
CD82	CXCR4	RPL27A	KLRB1	RPS10	FOSB	MT-ND2	BNIP3L	CD52	PLAC8

Table S3
List of 119 genes significantly overexpressed in T-ALL blasts (p value < .05, and $\geq$ 1.5-fold change).

	gene	avg_log2FC	pct.1	pct.2
1	MT-ND4L	3.65	0.99	0.49
2	MT-ATP8	3.10	0.97	0.18
3	SOX4	2.35	0.91	0.12
4	JUN	2.27	0.82	0.26
5	STMN1	2.23	0.86	0.24
6	TUBA1B	2.19	0.82	0.43
7	CD99	2.16	0.96	0.53
8	KLF6	2.13	0.86	0.25
9	HES4	2.11	0.54	0.02
10	FOS	2.02	0.62	0.16
11	EEF1G	2.02	0.93	0.27
12	TUBB	1.85	0.84	0.38
13	TUBA1A	1.80	0.85	0.25
14	PTPRCAP	1.74	0.75	0.02
15	GSTP1	1.66	0.85	0.34
16	MZB1	1.60	0.68	0.03
17	NME2	1.60	0.76	0.07
18	PPP1R15A	1.47	0.70	0.12
19	TAGLN2	1.47	0.89	0.44
20	CHI3L2	1.43	0.41	0.04
21	CDK6	1.43	0.68	0.07
22	SELENOW	1.43	0.81	0.35
23	GADD45B	1.37	0.57	0.17
24	IER2	1.34	0.79	0.45
25	ARMH1	1.33	0.67	0.16
26	MT-ND6	1.32	0.63	0.08
27	MARCKSL1	1.31	0.79	0.19
28	TXN	1.30	0.77	0.25
29	EIF4G2	1.28	0.79	0.26
30	AC084033.3	1.23	0.64	0.04
31	H1FX	1.21	0.66	0.29
32	GLUL	1.20	0.59	0.03
33	SLC25A5	1.19	0.86	0.45
34	DNTT	1.12	0.44	0.02
35	TUBB4B	1.12	0.55	0.08
36	HIST1H1E	1.08	0.46	0.03
37	PSMA6	1.08	0.60	0.09
38	SPTBN1	1.07	0.58	0.09
39	YWHAZ	1.07	0.88	0.44
40	RHOA	1.06	0.91	0.53
41	GABARAP	1.02	0.68	0.12
42	CLIC1	1.02	0.89	0.48
43	CAPG	1.01	0.53	0.05
44	SRSF10	1.01	0.69	0.22
45	H2AFY	1.00	0.70	0.27
46	CD82	0.98	0.51	0.06
47	MIR181A1HG	0.98	0.49	0.02
48	GNA15	0.97	0.44	0.01
49	TPM4	0.95	0.49	0.11
50	PTP4A2	0.95	0.71	0.30
51	TFDP2	0.95	0.47	0.07
52	ATP6V0C	0.95	0.53	0.00
53	LDHA	0.94	0.78	0.45
54	PSAP	0.94	0.69	0.28
55	TKT	0.93	0.70	0.27
56	PGM2L1	0.92	0.43	0.04
57	MYO7B	0.91	0.37	0.00
58	44445.00	0.91	0.82	0.49
59	COX6A1	0.90	0.89	0.54
60	SEC61B	0.90	0.79	0.40

	gene	avg_log2FC	pct.1	pct.2
61	SFPQ	0.89	0.68	0.34
62	RNASEK	0.89	0.54	0.03
63	MBNL1	0.89	0.70	0.35
64	GALNT2	0.89	0.48	0.03
65	ADA	0.88	0.52	0.09
66	ALDOA	0.83	0.66	0.28
67	HIST1H1D	0.83	0.39	0.03
68	WSB1	0.81	0.55	0.20
69	RBBP6	0.81	0.52	0.11
70	BRD2	0.81	0.55	0.13
71	ATP5MC3	0.81	0.75	0.45
72	ATP5F1B	0.80	0.77	0.43
73	TPM3	0.79	0.83	0.51
74	SPTAN1	0.78	0.49	0.08
75	EPC1	0.77	0.69	0.32
76	RCBTB2	0.77	0.39	0.04
77	RCC1	0.75	0.43	0.04
78	POLR2A	0.75	0.45	0.07
79	NREP	0.75	0.40	0.01
80	CLEC11A	0.75	0.37	0.04
81	SSH2	0.74	0.46	0.09
82	H2AFV	0.74	0.64	0.29
83	LST1	0.72	0.43	0.10
84	TASP1	0.72	0.36	0.03
85	HIST1H1C	0.71	0.36	0.05
86	CCNL1	0.71	0.66	0.35
87	VAT1	0.71	0.40	0.02
88	FDFT1	0.71	0.47	0.14
89	RUNX1	0.71	0.43	0.05
90	STT3B	0.71	0.49	0.15
91	KHDRBS1	0.70	0.68	0.34
92	CHD1	0.70	0.44	0.11
93	HNRNPL	0.69	0.48	0.12
94	SET	0.69	0.71	0.41
95	IDH2	0.67	0.54	0.21
96	SLC38A2	0.67	0.46	0.13
97	RAD21	0.66	0.53	0.19
98	ZNF428	0.66	0.59	0.22
99	SRSF11	0.65	0.63	0.32
100	RALY	0.65	0.64	0.28
101	C9orf16	0.65	0.76	0.45
102	JPT1	0.65	0.52	0.18
103	SNHG15	0.65	0.44	0.11
104	DDAH2	0.63	0.40	0.07
105	COX5A	0.63	0.66	0.32
106	RAB37	0.63	0.39	0.08
107	ILF2	0.62	0.55	0.22
108	ELOVL5	0.62	0.48	0.14
109	SCAI	0.61	0.39	0.06
110	MATR3.1	0.61	0.39	0.02
111	EWSR1	0.61	0.55	0.23
112	VCP	0.60	0.50	0.18
113	HP1BP3	0.60	0.53	0.22
114	AIF1	0.60	0.61	0.28
115	PRDX5	0.60	0.64	0.32
116	SF3B2	0.59	0.67	0.36
117	ARF1	0.59	0.71	0.41
118	ITGAE	0.58	0.43	0.10
119	YWHAQ	0.58	0.62	0.31

Table S4

Univariate and multivariate analysis of event-free survival association of the top T-ALL blast overexpressing genes using the TARGET ALL Phase2 T cell cohort. Significant p-values ($p < 0.05$) are marked with asterisk. Hazard ratios (HRs) were calculated with low expression as reference for gene-based survival associations, and MRD negative or CNS1 (CNS Negative) as references for clinical-based survival associations. Multivariate analysis was performed with all 24 genes shown, MRD status, and CNS status as variants.

Gene name	Grouping method: cutpoint (cutpoint | P-value | percentile)	Univariate			Multivariate (gene expression + MRD status + CNS)	
		Defined groups #Low | #High	Hazard Ratio **HR (95% CI)**	Log Rank p-value| HR p-value	HR (95% CI)	HR p-value
MT-ND4L	*7.66 | 0.700 | 0.556*	*145 | 116*	*0.60 (0.27-1.30)*	*0.21 | 0.21*	*1.296 (0.297-5.66)*	*0.73*
MT-ATP8	*5.21 | 0.661 | 0.333*	*87 | 174*	*0.57 (0.27-1.20)*	*0.14 | 0.15*	*0.465 (0.101-2.13)*	*0.325*
SOX4	*7.50 | 0.777 | 0.460*	*120 | 141*	*1.40 (0.65-3.10)*	*0.39 | 0.39*	*1.415(0.371 – 5.40)*	*0.611*
JUN	*7.72 | 0.520 | 0.322*	*84 | 177*	*2.20 (0.82-5.70)*	*0.11 | 0.12*	*1.462 (0.349 – 6.12)*	*0.603*
STMN1	*7.25 | 0.859 | 0.732*	*191 | 70*	*1.50 (0.67-3.30)*	*0.33 | 0.33*	*10.488(1.473 – 74.67)*	*0.019**
TUBA1B	*7.00 | 0.750 | 0.437*	*114 | 147*	*0.62 (0.29-1.30)*	*0.21 | 0.21*	*0.096(0.014 – 0.65)*	*0.016**
CD99	*7.43 | 0.277 | 0.387*	*101 | 160*	*2.20 (0.91-5.60)*	*0.072 | 0.0804*	*0.647 (0.129 – 3.24)*	*0.597*
KLF6	*7.32 | 0.717 | 0.739*	*193 | 68*	*0.47 (0.16-1.4)*	*0.16 | 0.17*	*0.509(0.095 – 2.74)*	*0.432*
HES4	*3.43 | 0.0857 | 0.254*	*67 | 194*	*4.50 (1.10-19.0)*	*0.025 | 0.041**	*4.670(0.885 – 24.65)*	*0.069*
FOS	*7.84 | 0.140 | 0.774*	*202 | 59*	*0.12 (0.016-0.89)*	*0.013 | 0.038**	*0.0117(0.013 – 1.10)*	*0.06*
EEF1G	*0.352 | 0.859 | 0.203*	*56 | 205*	*0.58 (0.26-1.30)*	*0.18 | 0.19*	*0.319 (0.094 – 1.08)*	*0.067*
TUBB	*7.24 | 0.914 | 0.169*	*44 | 217*	*2.60 (0.62-11.0)*	*0.17 | 0.19*	*1.045 (0.108 – 10.10)*	*0.97*
TUBA1A	*7.32 | 0.536 | 0.686*	*179 | 82*	*0.47 (0.18-1.30)*	*0.12 | 0.13*	*0.396 (0.077 – 2.04)*	*0.268*
GSTP1	*8.02 | 0.812 | 0.885*	*231 | 30*	*2.40 (0.95-5.90)*	*0.058 | 0.065*	*1.685(0.356 – 7.96)*	*0.511*
MZB1	*6.89 | 0.0261 | 0.479*	*125 | 136*	*2.70 (1.10-6.40)*	*0.018 | 0.024**	*1.797 (0.488 – 6.61)*	*0.378*
NME2	*0.815 | 0.490 | 0.597*	*159 | 102*	*1.80 (0.86-3.90)*	*0.11 | 0.11*	*5.16 (1.444 – 18.44)*	*0.012 **
PPP1R15A	*7.64 | 0.450 | 0.483*	*126 | 135*	*0.54 (0.25-1.20)*	*0.12 | 0.13*	*1.319 (0.354 – 4.91)*	*0.68*
TAGLN2	*7.74 | 0.913 | 0.870*	*227 | 34*	*0.25 (0.034-1.80)*	*0.14 | 0.17*	*0.222(0.023-2.17)*	*0.196*
CHI3L2	*7.33 | 0.307 | 0.759*	*198 | 63*	*2.20 (1.00-4.80)*	*0.035 | 0.040**	*1.074 (0.321-3.60)*	*0.908*
CDK6	*7.68 | 0.178 | 0.716*	*187 | 74*	*2.30 (1.10-4.90)*	*0.024 | 0.029**	*4.448 (1.439-13.75)*	*0.01***
SELENOW(SEPW1)	*3.90 | 0.205 | 0.218*	*57 | 204*	*3.80 (0.89-16.0)*	*0.053 | 0.072*	*1.280 (0.184 – 8.89)*	*0.803*
GADD45B	*5.23 | 0.94 | 0.322*	*84 | 177*	*0.66 (0.31-1.40)*	*0.29 | 0.29*	*0.349(0.126-0.97)*	*0.043**
IER2	*4.93 | 0.787 | 0.295*	*77 | 184*	*1.90 (0.72-5.00)*	*0.19 | 0.19*	*8.540(2.084-34.99)*	*0.003***
ARMH1(C1orf228)	*5.92 | 0.094 | 0.686*	*179 | 82*	*2.50 (1.20-5.40)*	*0.012 | 0.016**	*6.800 (2.266 – 20.41)*	*<0.001****
MRD_Day29_split					*1.727(0.630-4.74)*	*0.288*
CNS2					*0.848(0.241-2.99)*	*0.798*
CNS3					*7.698 (2.128 – 27.85)*	*0.002***
